# Proposal for a room-temperature diamond maser

**DOI:** 10.1038/ncomms9251

**Published:** 2015-09-23

**Authors:** Liang Jin, Matthias Pfender, Nabeel Aslam, Philipp Neumann, Sen Yang, Jörg Wrachtrup, Ren-Bao Liu

**Affiliations:** 1 Department of Physics and Centre for Quantum Coherence, The Chinese University of Hong Kong, Shatin, New Territories, Hong Kong, China; 2 3rd Institute of Physics, University of Stuttgart, 70569 Stuttgart, Germany

## Abstract

The application of masers is limited by its demanding working conditions (high vacuum or low temperature). A room-temperature solid-state maser is highly desirable, but the lifetimes of emitters (electron spins) in solids at room temperature are usually too short (∼ns) for population inversion. Masing from pentacene spins in *p*-terphenyl crystals, which have a long spin lifetime (∼0.1 ms), has been demonstrated. This maser, however, operates only in the pulsed mode. Here we propose a room-temperature maser based on nitrogen-vacancy centres in diamond, which features the longest known solid-state spin lifetime (∼5 ms) at room temperature, high optical pumping efficiency (∼10^6^ s^−1^) and material stability. Our numerical simulation demonstrates that a maser with a coherence time of approximately minutes is feasible under readily accessible conditions (cavity *Q*-factor ∼5 × 10^4^, diamond size ∼3 × 3 × 0.5 mm^3^ and pump power <10 W). A room-temperature diamond maser may facilitate a broad range of microwave technologies.

## Abstract (web summary)

Maser applications are hindered by their demanding working conditions. Here, Jin *et al*. theoretically propose a room-temperature maser based on nitrogen vacancy centres in diamond. This numerical study demonstrates that the operation of the maser under readily accessible conditions is feasible.

## Graphical abstract

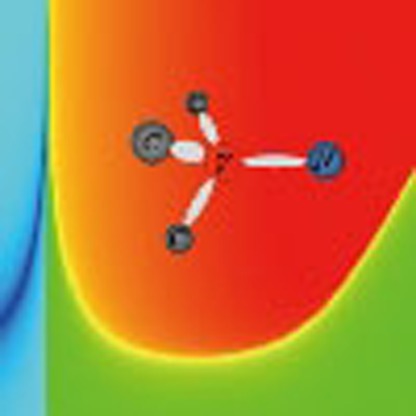

The maser^1^—with a frequency 0.3–300 GHz and a wavelength between 1 m and 1 mm—is the microwave analogue of lasers with several important applications^2^,^3^,^4^,^5^ such as in ultrasensitive magnetic resonance spectroscopy, astronomy observation, space communication, radar and high-precision clocks. Such applications, however, are hindered by the demanding operation conditions (high vacuum for a gas maser^6^ and liquid-helium temperature for a solid-state maser^7^). Room-temperature solid-state masers are highly desirable.

The key to a maser is the population inversion of the emitters and the macroscopic coherence among microwave photons. Population inversion requires a spin relaxation rate lower than the pump rate. This sets the bottleneck in room-temperature solid-state masers, as the spin relaxation times in solids are usually extremely short (approximately nanoseconds^8^) at room temperature due to rapid phonon scattering^7^. The spin relaxation induced by phonon scattering can be largely suppressed in light-element materials (such as organic materials) where the spin–orbit coupling is weak. Actually, the only room-temperature solid-state maser demonstrated so far is based on a pentacene-doped *p*-terphenyl molecular crystal where the spin lifetime can reach 135 μs at room temperature^9^. However, the active spins in pentacene-doped *p*-terphenyl are the intermediate metastable states instead of the ground states. Such an energy level structure greatly reduces the optical pumping efficiency and the pentacene maser requires high pump laser power. In addition, the organic material of *p*-terphenyl molecular crystal is unstable under high-power pump and at high temperature. The pentacence maser works only at the pulsed mode with a quite low repetition rate (∼1 Hz)^9^. Another candidate system under consideration is the silicon vacancy (V_Si_) centre in silicon carbide^10^. Very recently, it has been clarified that the V_Si_ centre has a spin-3/2 ground state, which allows population inversion by an optical pump^10^,^11^. The spin lifetime is quite long (∼0.5 ms) at room temperature^10^,^11^. Stimulated microwave emission from V_Si_ centres has been observed at room temperature^10^. Masing from the silicon carbide spins, however, is still elusive. The challenges include the complexity of the defects in the compound material and the difficulty of engineering the V_Si_ centres.

Nitrogen-vacancy (NV) centre spins in diamond^12^ have been extensively studied for quantum information processing^12^ and quantum sensing^13^,^14^,^15^. This is due to their long coherence time at room temperature and high efficiencies of initialization by optical pumping and readout via photon detection. In particular, coupling between ensemble NV centre electron spins and superconducting resonators has been demonstrated for quantum information storage and retrieval in hybrid quantum systems at cryogenic temperature^16^,^17^. Enhanced quantum coherence of NV centre ensembles has been observed in the strong coupling regime at low temperature^18^. It should be noted that the superb spin coherence features of NV centres persist at room temperature and even at temperatures up to at least 600 K (ref. 19), and the requirement of low temperatures in refs 16, 17, 18 is mostly for the resonators used. On the basis of these works, we here propose a new class of quantum technologies based on NV centres in diamond, namely, room-temperature solid-state masers and microwave amplifiers.

NV centres in diamond possess all of the features needed for a room-temperature solid-state maser. NV centre spins have the longest known lifetime (∼5 ms) at room temperature^20^,^21^,^22^ among all of the solid-state spins (∼50 times longer than ∼0.1 ms in pentacene-doped *p*-terphenyl^9^ and ∼10 times longer than ∼0.5 ms in silicon carbide^10^,^11^). The spin is a triplet (spin-1) in the ground state and it can be optically pumped rapidly to the *m*_s_=0 ground state, with a pump rate as high as ∼10^6^ s^−1^ (refs 23, 24, 25, 26) (versus ∼10^3^ to 10^5^ s^−1^ in organic materials^9^ and ∼10^5^ s^−1^ in silicon carbide^27^). Therefore, the population inversion can be easily achieved if a magnetic field is applied to shift the *m*_s_=0 ground state to above another spin state. Furthermore, the good thermal conductivity and material stability of diamond are also advantageous for masers. All of these features suggest that the NV centres in diamond are a superb gain medium for a room-temperature solid-state maser. However, the diamond maser requires a magnetic field; this is a further experimental complication that can, however, be overcome because magnets that can provide stable and uniform magnetic fields are commercially available^28^,^29^,^30^.

Here, we theoretically propose a room-temperature maser based on NV centres in diamond. Our numerical simulation demonstrates that masing and microwave amplification are feasible under readily accessible conditions (cavity *Q*-factor ∼50,000, diamond size ∼3 × 3 × 0.5 mm^3^ and NV centre concentration ∼2 p.p.m.), with the 532-nm optical pump threshold ∼3 W for microwave amplifying and ∼4 W for masing. For a pump power <10 W, it is feasible to achieve masing with an output power of >10 nW, a coherence time of approximately minutes, an mHz linewidth and a sensitivity of <10 pT Hz^−1/2^ for magnetometry application. As a room-temperature microwave amplifier, the noise temperature is as low as ∼0.3 K under a few-watt pump. A room-temperature diamond maser may facilitate a broad range of microwave technologies.

## Results

### System and model

**Figure 1:**
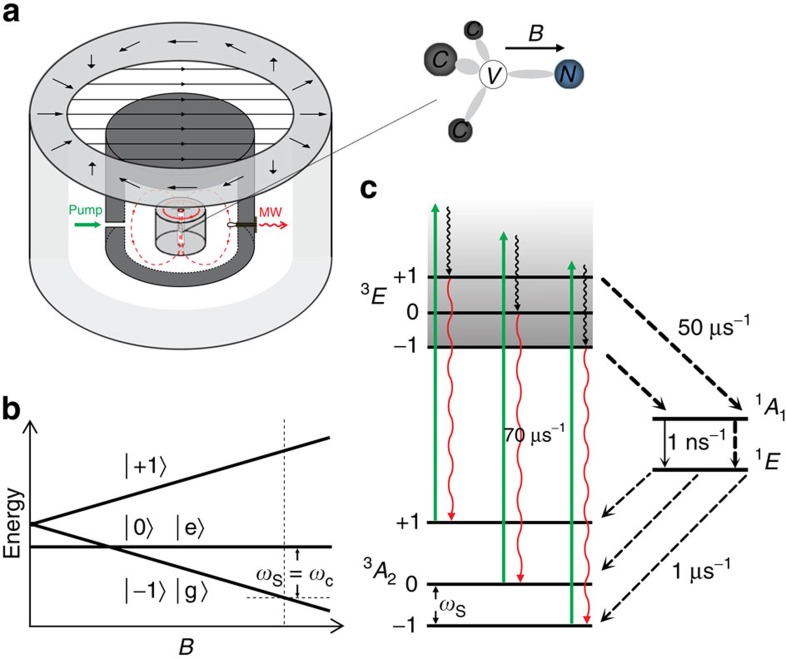
Scheme of room-temperature diamond maser. (**a**) The diamond maser system. A diamond sample is fixed inside a high-quality sapphire microwave dielectric resonator loaded in a coaxial cylindrical cavity. Microwave signal outputs from the loop coupling to the TE_01δ_ mode magnetic field (dashed red circles). The Halbach magnet array (the outer cylindrical wall) provides a uniform external magnetic field along the NV axis, which is set perpendicular to the cavity axial direction. The NV centres are pumped by a 532-nm light (green arrow). (**b**) The energy levels of an NV centre spin as functions of a magnetic field *B*. The zero-field splitting at *B*=0 is about 2.87 GHz. The magnetic field is set such that the transition frequency *ω*_S_ between the states 
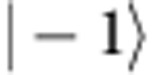
 
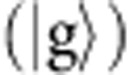
 and 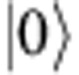 
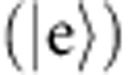
 is resonant with the cavity mode frequency *ω*_c_. (**c**) The pump scheme. After the optical excitation by a 532-nm light (green arrows), the excited-state ^3^*E* can directly return to the ground-state ^3^*A*_2_ via spin-conserving photon emission at a rate of ∼70 μs^−1^, but the excited states 
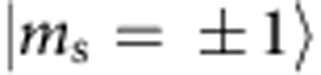
 can also decay to the singlet state ^1^*A*_1_ via inter-system crossing at a rate of ∼50 μs^−1^ and quickly decay to the metastable state ^1^*E*, then relax back to the three different ground states at a rate of ∼1 μs^−1^ in each pathway.

We consider an ensemble of NV centre spins in diamond resonantly coupled to a high-quality microwave sapphire dielectric resonator cavity^9^,^31^ (Fig. 1a; Supplementary Note 1). This type of cavity has been experimentally demonstrated to have a *Q*-factor of >10^5^ at room temperature^9^,^31^. Note that many other types of microwave resonators^10^,^32^ may also be considered for implementing the proposal in this paper. The spin sublevels 
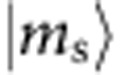
 (*m*_s_=0 or *m*_s_=±1) of the NV triplet ground state have a zero-field splitting about 2.87 GHz between 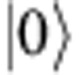 and 
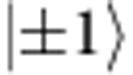
 (ref. 12) (Fig. 1b). The NV centres can be optically pumped to the state 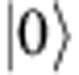 (ref. 12). A moderate external magnetic field (>1,000 G) splits the states 
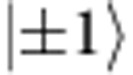
 and shifts the 
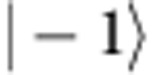
 state to below 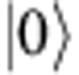 so that the spins can be inverted by an optical pump (Fig. 1b,c). The transition frequency *ω*_S_ between the spin ground state 
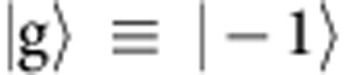
 and the spin exited state 
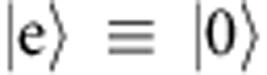
 is tuned resonant with the microwave cavity frequency *ω*_c_.

The maser is driven by coupling between the cavity mode and the spins. The Hamiltonian of the coupled spin–cavity system is 
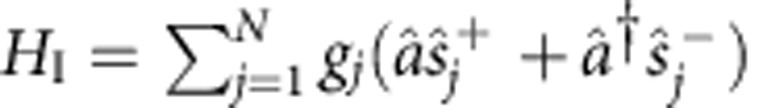
, where 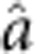 annihilates a microwave cavity photon, 
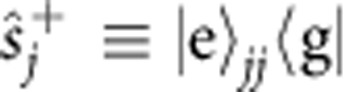
 is the raising operator of the *j*-th spin, 
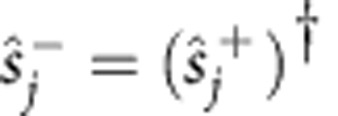
, and *g*_*j*_ is the coupling constant. Without changing the essential results, we assume that the spin–photon coupling is uniform, that is, *g*_*j*_=*g* and write the Hamiltonian as 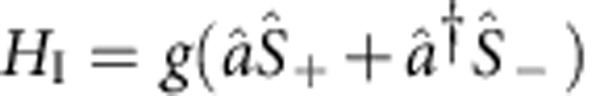 with the collective operators 
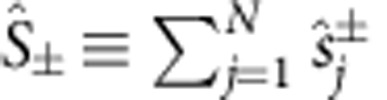
, which satisfy the commutation relation 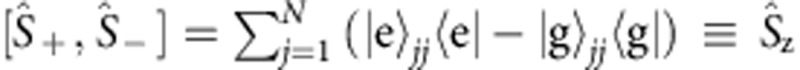. When masing occurs, the spin polarization (or population inversion) 
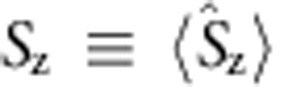
 is a macroscopic number 
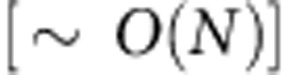
, while the fluctuation 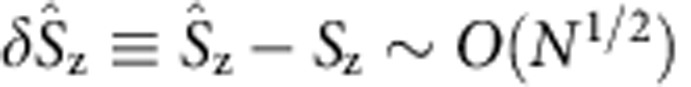 is much smaller. Therefore, 
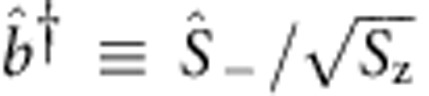
 can be interpreted as the creation operator of a collective mode with 
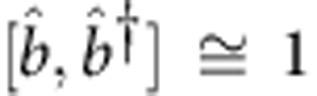
. The creation operator generates coherent superposition states in the spin ensemble. For example, from a fully polarized spin state, the collective mode state excited by one cavity photon is a quantized spin wave 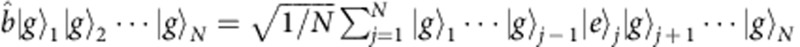, which acts as a boson. In the masing state, both the photons and the spin collective modes, coherently coupled to each other, have macroscopic amplitudes. With the excitation number of the coherent spin collective mode 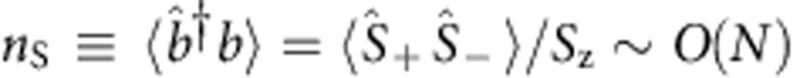, the spins are in a macroscopic quantum superposition state, which is maintained by the masing process.

A prerequisite of masing is the spin population inversion. The optical pumping rate *w* can be tuned by varying the pump light intensity, up to ∼10^6^ s^−1^ (refs 23, 24, 25, 26). The cavity mode has a decay rate determined by the cavity *Q*-factor, *κ*_c_=*ω*_c_/*Q*, due to photon leakage and coupling to the input/output channels. The decay of the spin collective mode is caused by various mechanisms. First, the spin relaxation (*T*_1_ process caused by phonon scattering and resonant interaction between spins) contributes a decay rate *γ*_eg_=1/*T*_1_. Second, the individual spins experience local field fluctuations due to interaction with nuclear spins, coupling to other NV and nitrogen (P1) centre electron spins, and fluctuation of the zero-field splitting^17^. Such local field fluctuations induce random phases to individual spins, making the bright collective mode decay to other modes at a rate 
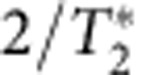
, where 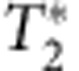 is the dephasing time of the spin ensemble. Finally, the optical pump of the NV centres, being incoherent, also induces the decay of the collective mode. The collective mode decay rate induced by the incoherent pump is *qw*, where *q*≈16 is an amplification factor of the contribution of pump rate *w* to the collective mode decay rate *κ*_S_ (see Supplementary Note 1), due to multiple pathways (Fig. 1c) of the pump process. The total decay rate of the collective mode is thus 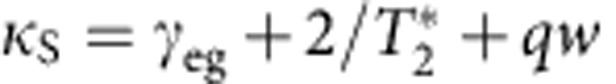. The coupled quantum dynamics of the collective modes and the photons is described by the quantum Langevin equations^33^ for the photon and spin collective mode operators 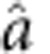 and 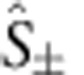, the spin populations 
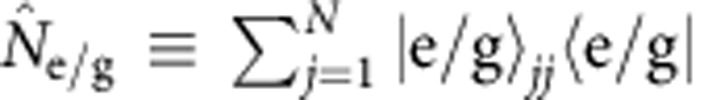
, and polarization 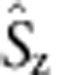 (see Methods and Supplementary Note 2).

For a specific system, we consider a single crystal bulk diamond of volume *V*_NV_=3 × 3 × 0.5 mm^3^ with a natural abundance (1.1%) of ^13^C nuclear spins, a P1 centre concentration of about 20 p.p.m. and an NV centre concentration of 2 p.p.m. (for N-to-NV^−^ conversion efficiency 10%). Such parameters are extracted from the diamond used in ref. 17. The ensemble spin decoherence time is 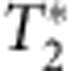
=0.4 μs (ref. 17) (see Supplementary Note 1). Considering the four orientations of NV centres and three nuclear spin states of ^14^N, the number of NV centres coupled to the cavity mode is estimated to be *N=ρ*_NV_*V*_NV_/12=1.32 × 10^14^. The external magnetic field 2,100 G results in *ω*_S_/2*π*≈3 GHz. The microwave dielectric resonator has its TE_01δ_ mode frequency resonant with the spin collective mode, that is, *ω*_c_=*ω*_S_. The cavity system (Fig. 1a) is composed of a cylindrical sapphire dielectric resonator (with radius *r*=15 mm and height *h*=16 mm), and a coaxial cylindrical cavity (with radius *R*=40 mm and height *H*=40 mm), placed inside a Halbach magnet array with a 50 (80)-mm inner (outer) radius, which provides the uniform magnetic field with inhomogeneity <0.01 G across the diamond size^28^,^29^. The coupling between the microwave photons and the NV centre spins is calculated to be *g*/2*π*≈0.02 Hz for the effective cavity mode volume^9^,^34^ *V*_eff_≈3 cm^3^. At room temperature (*T*=300 K), the phonon scattering dominates the spin relaxation and *γ*_eg_≈200 s^−1^ (refs 20, 21, 22). The number of thermal photons inside the cavity is *n*_th_≈2,100 at room temperature.

### Masing conditions

The quantum Langevin equations can be solved at steady-state masing. When masing occurs, the quantum operators can be approximated as their expectation values, that is, 
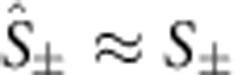
, 
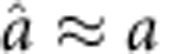
, 
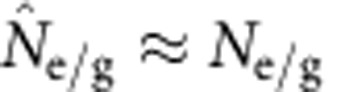
 and 
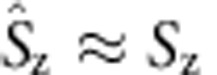
. By dropping the small quantum fluctuations, we reduce the quantum Langevin equations to classical equations for the expectation values (see Methods and Supplementary Note 2). Under the resonant condition (*ω*_c_=*ω*_S_), the steady-state solution is


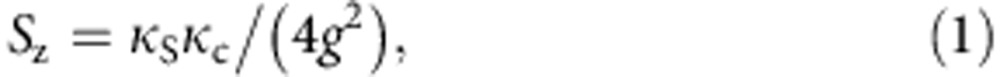



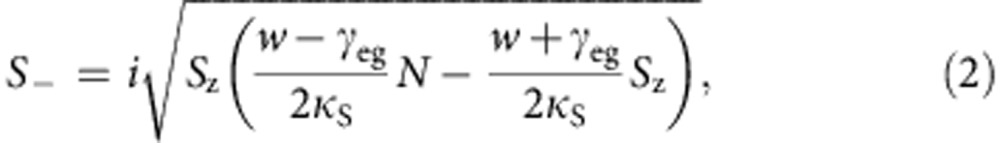



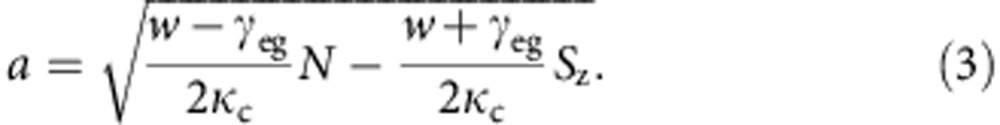


When the spin polarization *S*_z_∼*O*(*N*), we have *S*_+_*S*_−_∼*O*(*N*^2^). From equation (3), the number of intracavity photons 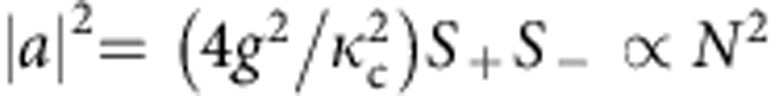 and consequently the output power 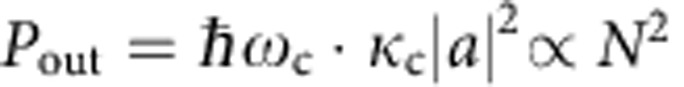, both scaling with the number of spins by *N*^2^.

The requirement that photon number 
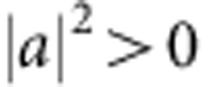
 leads to the masing condition


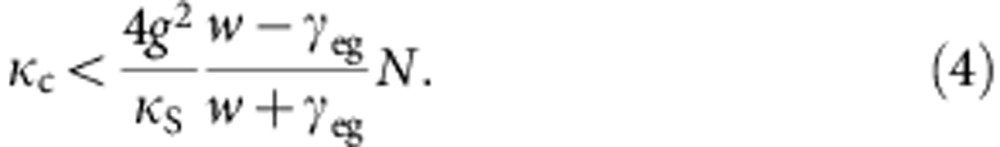


On one hand, the pump rate needs to be greater than the spin relaxation rate for population inversion (*w*>*γ*_eg_). On the other hand, the cavity *Q*-factor has to be above a threshold


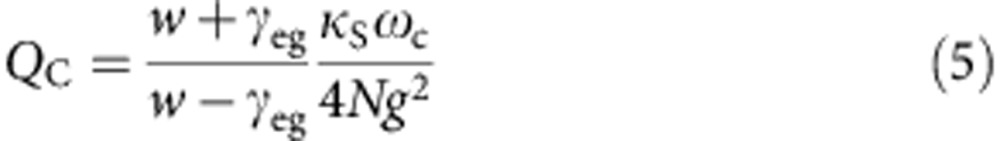


to have a sufficient number of photons for sustaining the macroscopic quantum coherence. A stronger spin–photon coupling (*g*), a smaller spin collective mode decay rate (*κ*_S_) or a larger number of spins (*N*) can reduce this threshold cavity *Q*-factor (see Supplementary Note 1 for more discussions). The cavity *Q*-factor threshold is equivalent to the requirement that the spin collective mode decay rate *κ*_S_ should be kept below the maximal collective emission rate of photons 4*Ng*^2^/*κ*_c_. Otherwise overpumping would fully polarize the spins, making the spin–spin correlation vanish (*S*_z_→*N* and *S*_−_→0).

**Figure 2:**
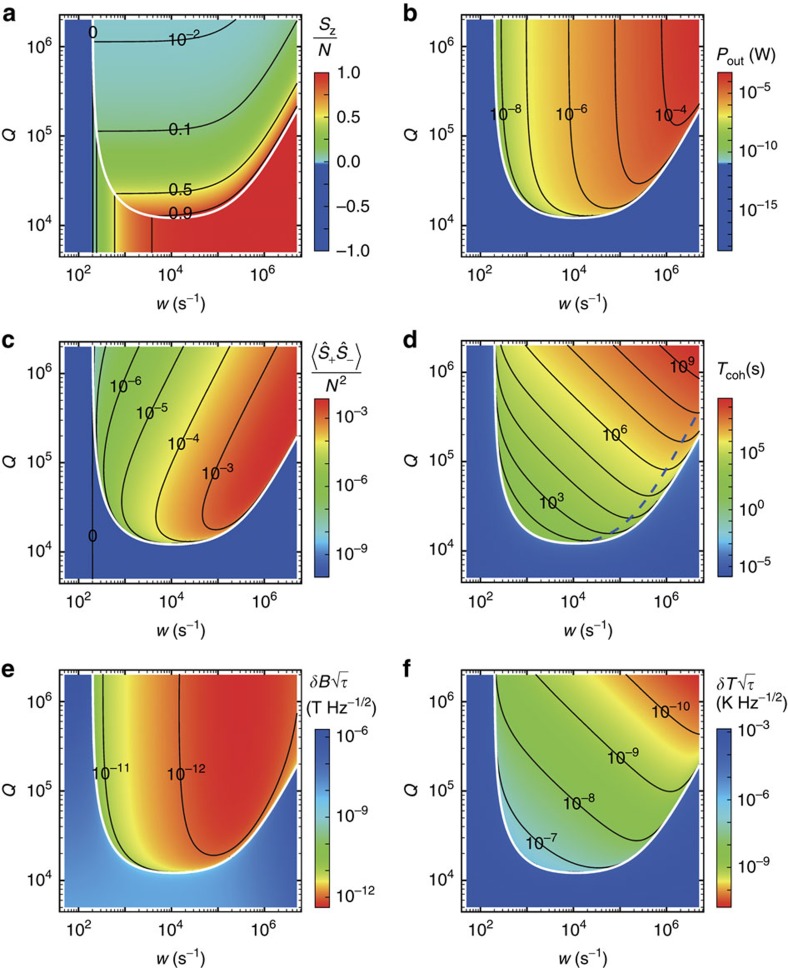
Room-temperature masing of NV centre spins in diamond. Contour plots of (**a**) the spin polarization *S*_z_, (**b**) the output power *P*_out_, (**c**) the collective spin–spin correlation 
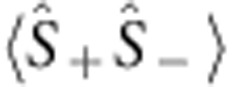
, (**d**) the macroscopic quantum coherence time *T*_coh_, (**e**) the sensitivity to external magnetic field and (**f**) the sensitivity to temperature, as functions of the pump rate *w* and the cavity *Q*-factor. The masing threshold is indicated in the figures by the white curves. The blue dashed curve in **d** shows the pump rate optimal for maximum coherence time. The parameters are such that *ω*_c_/2*π*=*ω*_S_/2*π*=3 GHz, *g*/2*π*=0.02 Hz, 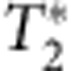
=0.4 μs, *N*=1.32 × 10^14^ and *γ*_eg_=200 s^−1^ at *T*=300 K.

The emergence of macroscopic quantum coherence is evidenced by macroscopic values of the spin–spin correlation, the photon number and the spin collective mode amplitude, and the long coherence time in the masing region (Fig. 2b–d). We calculated these quantities by using the higher-order equations of the correlation functions (see Methods and Supplementary Note 2), which apply to both masing and incoherent emission. The spin polarization *S*_z_, the microwave output power 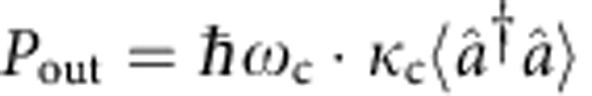 and the spin–spin correlation 
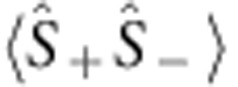
 (shown in Fig. 2a–c) are consistent with the results obtained from equation (2) when the pump rate and the cavity *Q*-factor are above the masing threshold (white curve in the Fig. 2). It is clearly seen that the output power increases markedly when the pump rate is above the spin relaxation rate (population inverted) and when the cavity *Q*-factor is above the masing threshold (*Q*>*Q*_C_) (Fig. 2b). The fact that 
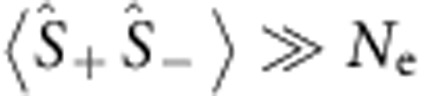
 unambiguously evidences the phase correlation among the large ensemble spins established by cavity photons in the masing region (Fig. 2c). The pump condition optimal for spin–spin correlation is determined by maximizing 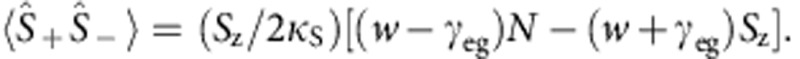 Under the condition that the pump is well above the threshold, 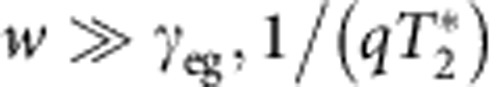, the spin–spin correlation reaches its maximum value 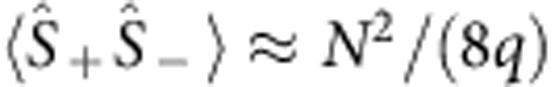 at the optimal pump rate 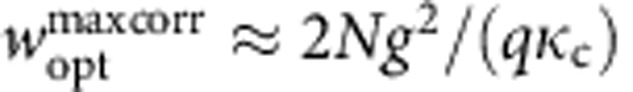, where the spin polarization *S*_z_≈*N*/2 and the maser power 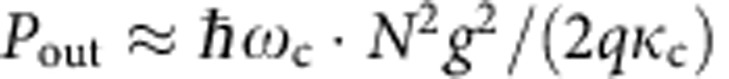 (see Supplementary Note 3).

The maser linewidth is determined by the correlation of the phase fluctuations of the photons or equivalently by that of the spin collective modes. The coherence time is obtained (see Supplementary Note 4) as


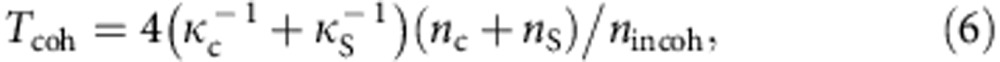


where 
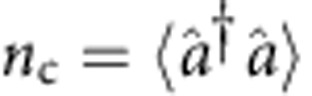
 is the photon number, 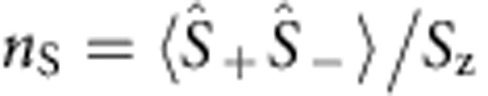 is the spin collective mode number and *n*_incoh_=*n*_th_+*N*_e_/*S*_z_ includes the thermal photon number (*n*_th_) and the incoherent spin collective mode number (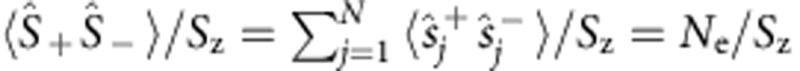 if the correlation between different spins is forced to be zero). The physical meaning of equation (6) is the following: the coherent excitations (photons and spin collective excitations) have the same phase within the total lifetime 
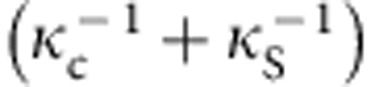
 of the photons and the spin collective modes; beyond the total lifetime, each incoherent excitation induces a random phase ∼*O*(*π*); the total random phase is shared by all the coherent excitations. Thus the random phase of a single photon or spin collective excitation accumulated during the total lifetime 
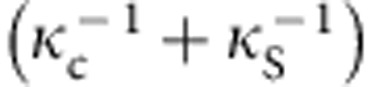
 is 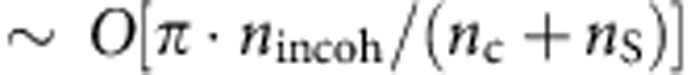. The coherence time is greatly enhanced in the masing region (Fig. 2d). The spin collective mode decay rate of the NV centres *κ*_S_>5 × 10^6^ s^−1^, while for a good microwave cavity (*Q*∼10^5^), the photon decay rate *κ*_c_∼6*π* × 10^4^ s^−1^, thus the photon number *n*_c_=*n*_S_*κ*_S_/*κ*_c_ is much greater than the spin collective mode number, and the macroscopic quantum coherence is mainly maintained by the photons in the cavity. For readily accessible cavities in experiments, the fractional frequency instability of a room-temperature diamond maser is ∼10^−12^*τ*^−1/2^ as listed in Tables 1 and 2 for a cavity with *Q*=5 × 10^4^. For comparison, the fractional frequency instability of a hydrogen maser is ∼10^−13^*τ*^−1/2^ at room temperature^35^ or ∼10^−15^*τ*^−1/2^ at cryogenic temperature^36^, and the state-of-the-art ytterbium atomic clock^37^ has a fractional frequency instability of ∼10^−16^*τ*^−1/2^. The optimal pump condition for a long coherence time can be obtained from equation (6). In the good-cavity or large ensemble limit where 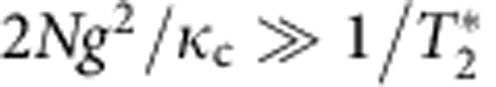 and at room temperature where *n*_incoh_≈*n*_th_, the optimal pump rate for maximum coherence time is close to that for maximum spin–spin correlation, that is, 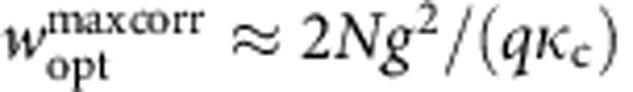, and the optimal coherence time reaches 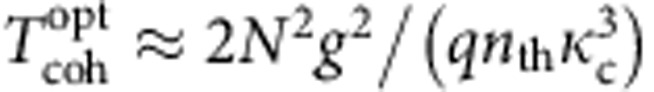, which scales with the spin number and the cavity *Q*-factor according to 
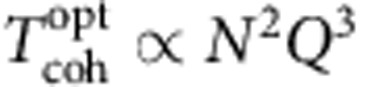
 (see Supplementary Note 3). Note that the quantum coherence sustained by active masing (with a pump) has a much longer lifetime than the spin coherence protected by passive coupling to the cavity (without a pump)^18^. This is due to the superradiant emission of photons from the spin collective modes of the NV centres in the bulk diamond and the concomitant large number of photons in the cavity.

### Diamond microwave amplifier

**Figure 3:**
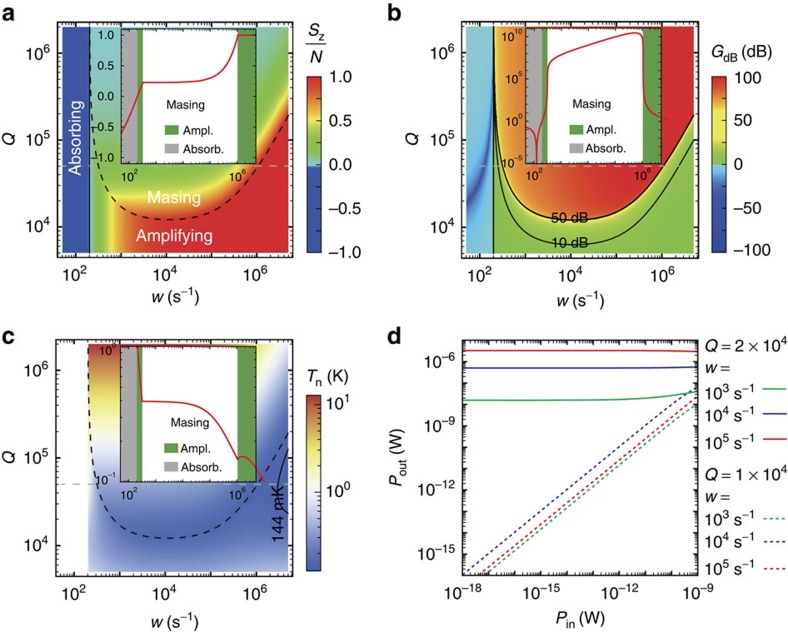
Room-temperature diamond microwave amplifier. Contour plots of (**a**) the spin polarization *S*_z_, (**b**) the power gain *G* and (**c**) the noise temperature *T*_n_, as functions of the pump rate *w* and the cavity *Q*-factor for resonant input microwave power *P*_in_=1 fW. For *w<γ*_eg_=200 s^−1^, the system is in the absorbing region (population not inverted). For *w>γ*_eg_=200 s^−1^ and the cavity *Q*-factor below/above masing threshold (the dashed curves), the system operates in the amplifying/masing mode. The insets show dependence on the pump rate for a fixed cavity *Q*=5 × 10^4^ (the absorbing, amplifying and masing regions marked as the grey, green and white, respectively). Amplifying is unstable in the masing region (see Supplementary Fig. 1). (**d**) Output power as a function of input microwave power for various pump rates (*w*=10^3^, 10^4^ or 10^5^ s^−1^), with the cavity *Q*-factor *Q*=10^4^ and 2 × 10^4^ corresponding to the amplifying and masing regions, respectively. In the amplifying regime (*Q*=10^4^), the amplification is linear in a large range of input power (see Supplementary Fig. 2 for more information). The parameters are the same as in Fig. 2.

The coupled spin–cavity system can be configured as a room-temperature microwave amplifier when the spin population is inverted (*S*_z_>0) but when the cavity *Q*-factor is below the masing threshold (*Q*<*Q*_C_). For the readily accessible parameter *Q*=10^4^, the noise temperature is as low as ∼0.2 K (versus ∼1 K for the state-of-the-art ruby amplifier working at liquid-helium temperature^3^). To study the amplification, we calculate the spin inversion, the microwave output power gain and the noise temperature with a weak microwave resonant input (see Methods and Supplementary Note 5). As shown in Fig. 3, the system linearly amplifies the microwave signal. The gain is about 6–10 dB under a 6–10-W pump with noise temperature 340–280 mK. At high pump around 136 W (*w=*10^4^ s^−1^), the gain is as high as 20 dB with noise temperature as low as 200 mK. The low noise temperatures indicate the single-photon noise level.

## Discussion

The ultralong coherence time of the maser is useful for metrology. The collective excitation of a large number of spins (∼10^14^) and cavity photons enhances the sensitivity. The sensitivity to a slow-varying magnetic field noise (with frequency ≤*κ*_S_/2) 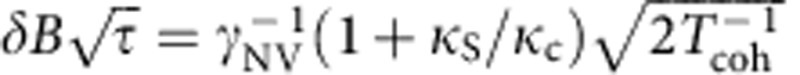 for measurement time *τ* (see Supplementary Note 6), where *γ*_NV_/2*π*=2.8 MHz G^−1^ is the NV centre gyromagnetic ratio. The magnetic field sensitivity is estimated to be in the order of 10–1 pT Hz^−1/2^ at room temperature (Tables 1 and 2). The temperature noise would induce cavity frequency fluctuation via thermal expansion and dielectric constant variation. The temperature sensitivity, 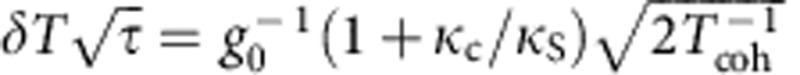 (see Supplementary Note 6), is estimated to be in the order of 100–10 nK Hz^−1/2^ at room temperature (Tables 1 and 2), where we have taken *g*_0_≈(*α*+*β/*2)*ω*_c_=165 kHz K^−1^ with *α* and *β* being the temperature coefficients of thermal expansion and permittivity for sapphire, respectively (see Supplementary Note 1). For a higher cavity *Q*-factor, the thermometry sensitivity is enhanced while the magnetometry sensitivity is reduced (see Fig. 2e,f) due to the frequency dragging effect (the steady-state masing frequency is a weighted average of the spin and cavity frequency^38^, *ω*=(*κ*_c_*ω*_S_+*κ*_S_*ω*_c_)/(*κ*_c_+*κ*_S_), see Supplementary Note 2). The sensitivities to the magnetic field and the temperature noises set the requirements on stability of the set-up for maintaining the long coherence time of the maser.

The pump power 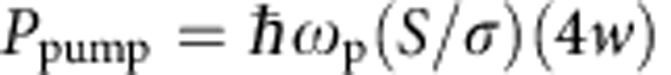 is proportional to the pump light frequency (*ω*_p_), the pump rate (*w*) and the area of the pump light spot (*S*) divided by the NV centre absorption cross-section (*σ*) (see Supplementary Note 1). The threshold pump power is low because the spin relaxation time is long in diamond. For a readily accessible cavity with *Q*=5 × 10^4^, we show diamond maser performance in Tables 1 and 2. The threshold pump power for microwave amplifying (population inversion) is estimated to be 2.7 W, above which the net photon emission into the cavity amplifies the signal. The threshold pump power for masing is estimated to be 4.3 W for *Q*=5 × 10^4^ cavity (see Supplementary Note 1 for pump thresholds), above which the emitted photons show collective coherence and the photon number scales with the spin number (*N*) by *n*_c_∝*N*^2^.

The optical pump may heat the system, inducing both temperature increase and fluctuation. The frequency shifts of the spins and the cavity due to temperature increase are not an issue since once the steady-state is reached, the spin transition frequency can be tuned to resonance with the cavity mode by tuning the magnetic field.

It has been demonstrated that NV centre spins still have good coherence properties at least up to 600 K (ref. 19). According to ref. 19, the spin polarization, the pump rate and the 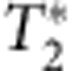 decoherence time are only slightly changed at a temperature as high as 650 K. The longitudinal spin relaxation time *T*_1_ is reduced to 0.34 ms at 600 K, more than 10 times shorter than that at room temperature, and the pump threshold for microwave amplifying (*w*>1/*T*_1_) is fulfilled when the pump power is >40 W. Also, the contribution of the longitudinal spin relaxation to spin collective mode decay is negligible 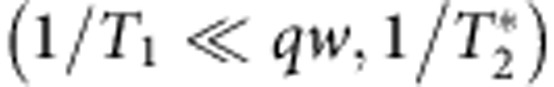.

**Figure 4:**
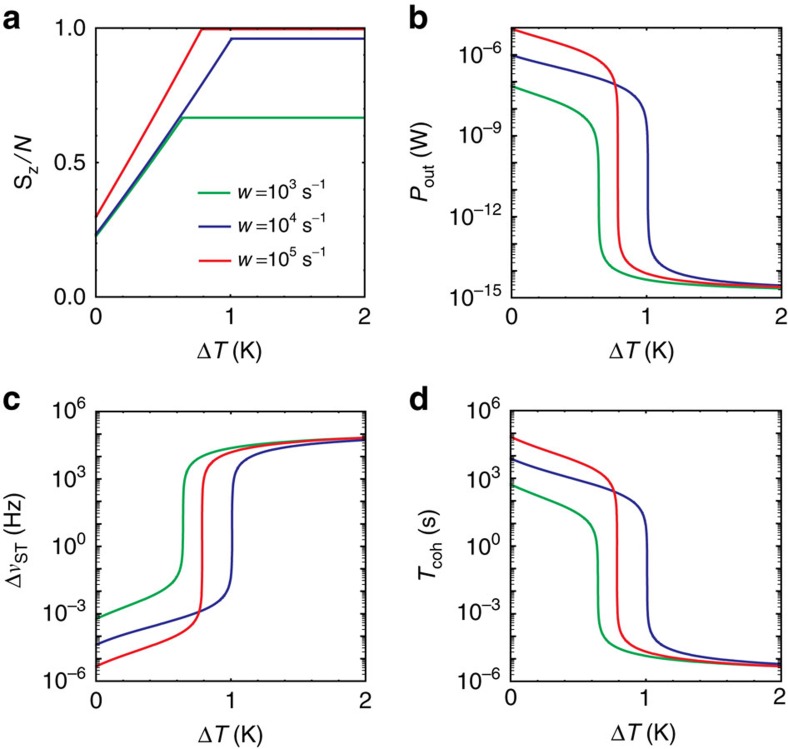
Temperature fluctuation effects on room-temperature masing. (**a**) The spin polarization, (**b**) the output power, (**c**) the linewidth and (**d**) the coherence time as functions of temperature fluctuation for cavity *Q*=5 × 10^4^, for various pump rates (*w*=10^3^, 10^4^ or 10^5^ s^−1^). The sharp changes indicate the transition between amplifying and masing. The parameters are the same as in Fig. 2.

The temperature fluctuation leads to transition frequency shifts of the spins (via lattice expansion) and the cavity mode (via dielectric constant variation and mode volume expansion), with (2*π*)^−1^Δ*ω*_S_/Δ*T*≈−74 kHz K^−1^ (ref. 39) and (2*π*)^−1^Δ*ω*_c_/Δ*T*≈−165 kHz K^−1^ (ref. 40) (see Supplementary Note 1). The masing conditions considered in this paper correspond to the photon leakage rate being much slower than the decay rate of the spin collective mode 
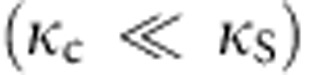
. Thus, the effects of temperature fluctuation on the maser result mainly from the cavity mode frequency fluctuation. Figure 4 shows the spin polarization, the output power, the masing linewidth and the coherence time as functions of the temperature fluctuation Δ*T* for cavity *Q*=5 × 10^4^, and various pump rates (*w*=10^3^, 10^4^, 10^5^ s^−1^). Note that the threshold cavity *Q*-factor is the lowest near *w*=10^4^ s^−1^ (Fig. 2), thus the rate near *w*=10^4^ s^−1^ is the most robust to temperature fluctuation. The masing condition is still fulfilled for up to 1.0 K temperature fluctuation at *w*=10^4^ s^−1^. However, temperature fluctuation does affect the maser performance. In Table 1 (2), we compare the maser performance with and without the 0.2 K (0.5 K) temperature fluctuation for *Q*=5 × 10^4^ cavity at low (high) pump power. For a larger number of spins, a larger temperature fluctuation can be tolerated (Δ*T*_max_∝*N*) due to the reduced threshold cavity *Q*-factor.

## Methods

### Maser equations

The theoretical study is based on the standard Langevin equations^33^


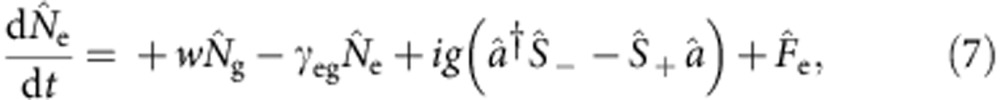



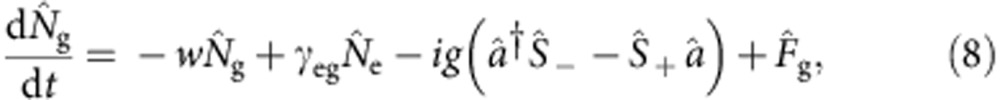



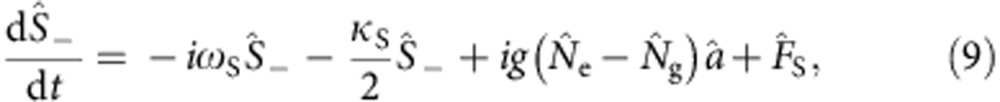



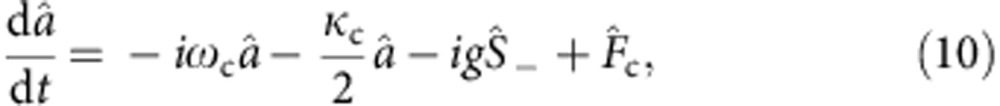


where 
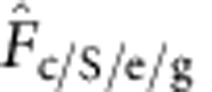
 is the noise operator that causes the decay of the photons (c), the spin collective modes (S), the population in the excited state (e) or that in the ground state (g). Note that the total spin number is written as an operator 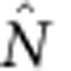 to take into account the fluctuation due to the population of the third spin state 
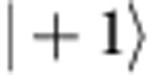
 and other intermediate states. The population fluctuation, however, has no effect on the phase fluctuation of the maser (see Supplementary Note 4).

By replacing the operators with their expectation values, we obtain the mean-field equations for the maser at the steady state


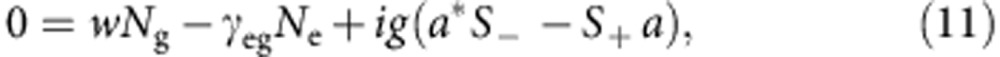



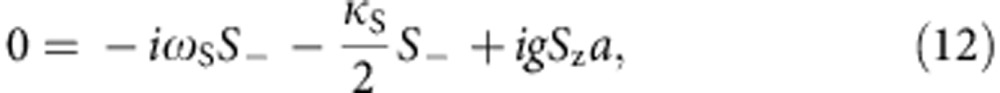



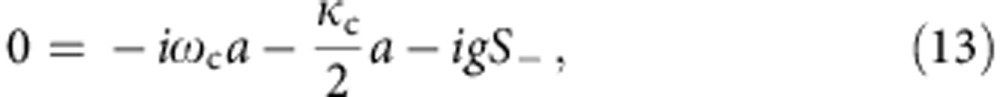


from which the masing frequency, the field amplitudes and the spin polarization can be straightforwardly calculated.

The coherence time and the linewidth are calculated using the spectrum of the phase fluctuations. The Langevin equations are linearized for the fluctuations, which are much smaller than the expectation values at steady state. The linearized equations are


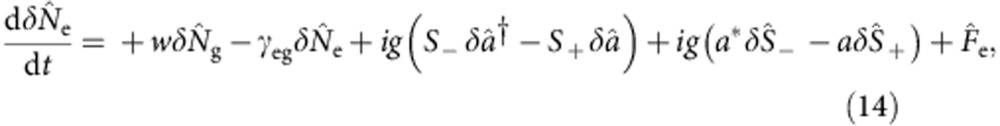



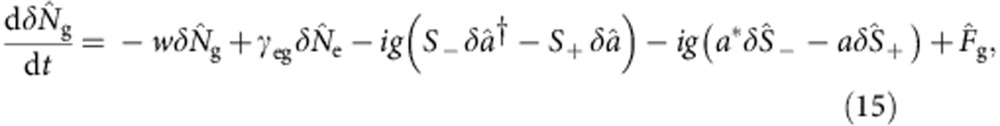



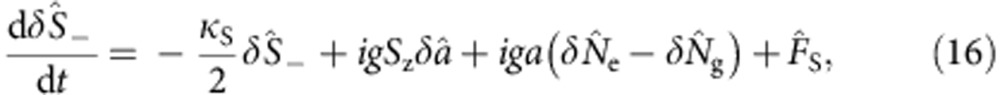



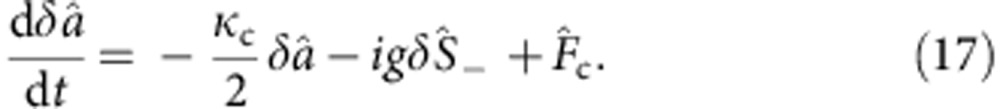


By Fourier transform of these equations, the spectrum of the phase noise can be calculated and hence the maser coherence time and linewidth are determined.

To investigate the correlations in both the masing and the incoherent emission regimes, we derive the equations for the correlation functions and take the expectation values of the relevant operators. That leads to


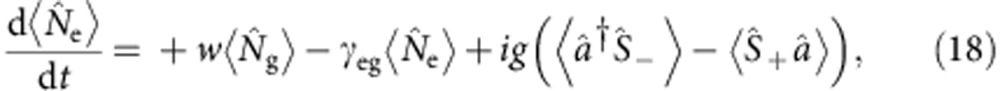



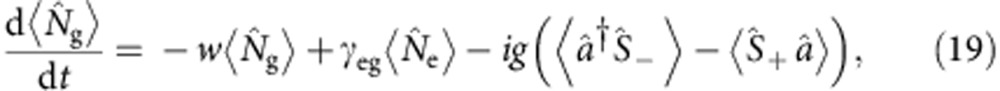



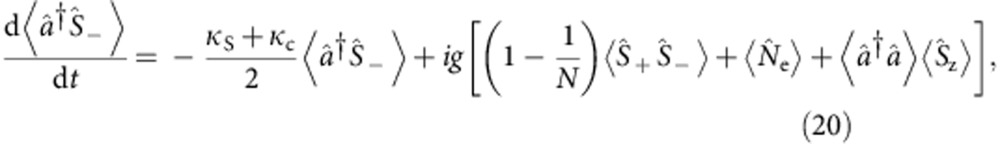



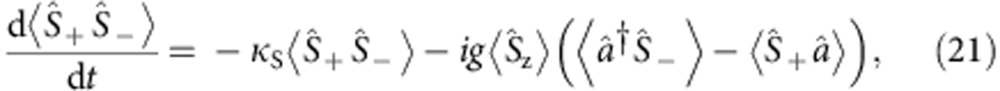



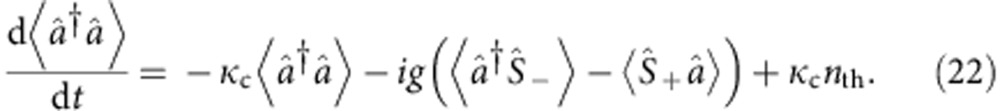


Here, to make the equations close, we have used the approximation 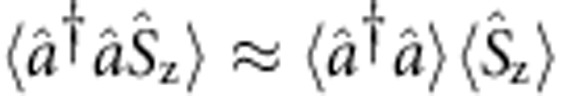, 
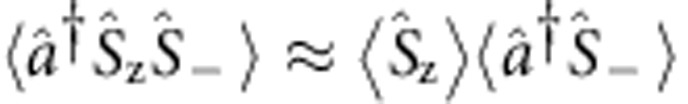
 and 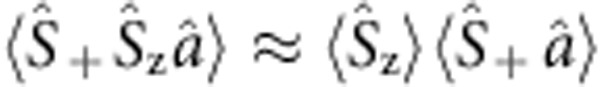, neglecting the higher-order correlations, which is well justified for Gaussian fluctuations.

### Microwave amplifier and noise temperature

To investigate the microwave amplifier, we solve the mean-field equations with a steady-state input 
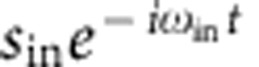
 as


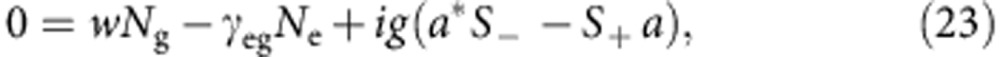



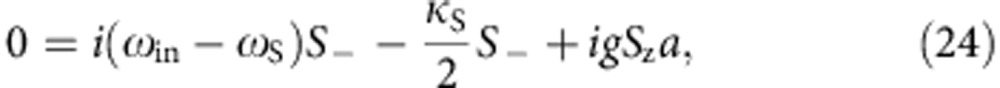



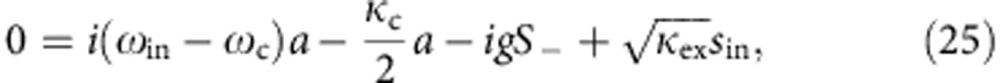



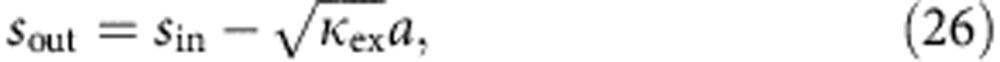


from which the power gain 
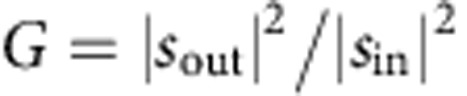
 is obtained. A power gain 
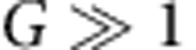
 is possible under the resonant condition *ω*_in_=*ω*_c_=*ω*_S_, but it will be reduced to *O*(1) at off-resonance, that is, 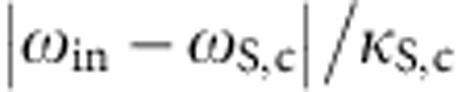
≳1. The intrinsic noise temperature^7^ of the diamond maser is given by


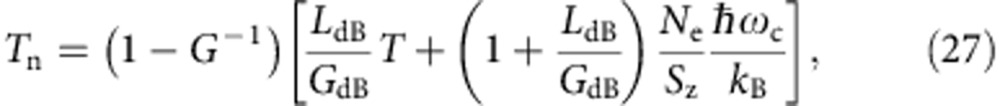


where 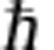 is the Planck constant, *k*_B_ is the Boltzmann constant, *T* is the environment temperature, *G*_dB_=10log_10_ *G* and 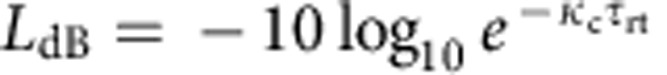 is the cavity power loss in decibel during the time of a microwave photon roundtrip time 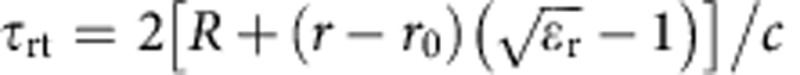 (*ɛ*_r_≈10 is the sapphire dielectric permittivity in the direction perpendicular to the *c* axis (resonator axis) of the sapphire crystal, *R* is the radius of the cylindrical cavity and *r* (*r*_0_) is the external (internal) radius of the dielectric resonator).

## Additional information

**How to cite this article:** Jin, L. *et al*. Proposal for a room-temperature diamond maser. *Nat. Commun.* 6:8251 doi: 10.1038/ncomms9251 (2015).

## Supplementary Material

Supplementary Information Supplementary Figures 1-2, Supplementary Note 1-6 and Supplementary References.

## Figures and Tables

**Table 1:** Performance of room-temperature diamond maser under low pump.

***P***_**pump**_ **(W)**	***w*** **(s**^−1^**)**	***P***_**out**_ **(nW)**	***T***_**coh**_ **(s)**	**Δ*****v***_**ST**_ **(mHz)**	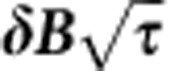 **(pT Hz**^−1/2^**)**	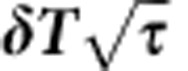 **(nK Hz**^−1/2^**)**	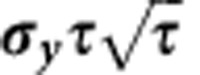 **(10**^−12^**)**
6.0 (1.0)	440.1 (440.1)	12.5 (1.0)	98 (5)	3.2 (61.1)	11.6 (34.3)	148.2 (666.2)	8.1 (36.6)
8.0 (1.4)	586.9 (586.9)	27.4 (8.9)	215 (49)	1.5 (6.5)	7.8 (11.2)	100.1 (218.1)	5.5 (12.0)
10.0 (1.7)	733.6 (733.6)	42.2 (16.9)	332 (92)	1.0 (3.5)	6.3 (8.2)	80.6 (158.6)	4.4 (8.7)

*P*_pump_, the pump power; *w*, the pump rate; *P*_out_, the output power; *T*_coh_, the coherence time; Δν_ST_, the maser linewidth; 
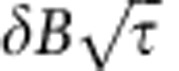
, the magnetic field sensitivity; 
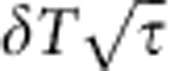
, the temperature sensitivity; 
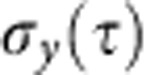
, the fractional frequency instability, where *τ* is the measurement time. The cavity *Q*=5 × 10^4^. The numbers in the brackets in the first column show the absorbed power *P*_absorb_, and the numbers in the brackets in other columns are values with a 0.2 K temperature fluctuation taken into account.

**Table 2:** Performance of room-temperature diamond maser under high pump.

***P***_**pump**_ **(W)**	***w*****(s**^−1^**)**	***P***_**out**_ **(μW)**	***T***_**coh**_ **(s)**	**Δ*****v***_**ST**_ **(μHz)**	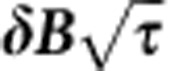 **(pT** **Hz**^−1/2^**)**	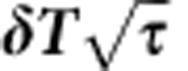 **(nK** **Hz**^−1/2^**)**	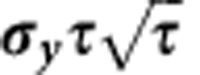 **(10**^−12^**)**
13.6 (2.4)	10^3^ (10^3^)	0.07 (0.007)	543 (27)	585.8 (11,891.6)	4.9 (10.7)	62.9 (308.6)	3.5 (17.0)
136.3 (23.7)	10^4^ (10^4^)	1.0 (0.2)	7,609 (836)	41.8 (380.6)	1.4 (2.0)	16.8 (55.0)	0.9 (3.0)
1,363.2 (236.7)	10^5^ (10^5^)	9.2 (1.5)	69,580 (5,312)	4.6 (59.9)	0.6 (1.0)	5.5 (21.2)	0.3 (1.2)

*P*_pump_, the pump power; *w*, the pump rate; *P*_out_, the output power; *T*_coh_, the coherence time; Δν_ST_, the maser linewidth; 
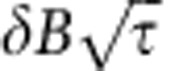
, the magnetic field sensitivity; 
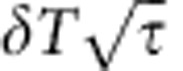
, the temperature sensitivity; 
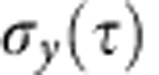
, the fractional frequency instability, where *τ* represents the measurement time. The cavity *Q*=5 × 10^4^. The numbers in the brackets in the first column show the absorbed power *P*_absorb_, and the numbers in the brackets in other columns are values with a 0.5 K temperature fluctuation taken into account.
