# Supplementary material for: Proposal for a room-temperature diamond maser
Source: Nat Commun. 2015 Sep 23;6:8251. doi: 10.1038/ncomms9251 (PMC4667537; doi:10.1038/ncomms9251)
Supplement: Supplementary Information — Supplementary Figures 1-2, Supplementary Note 1-6 and Supplementary References. [file ncomms9251-s1.pdf]

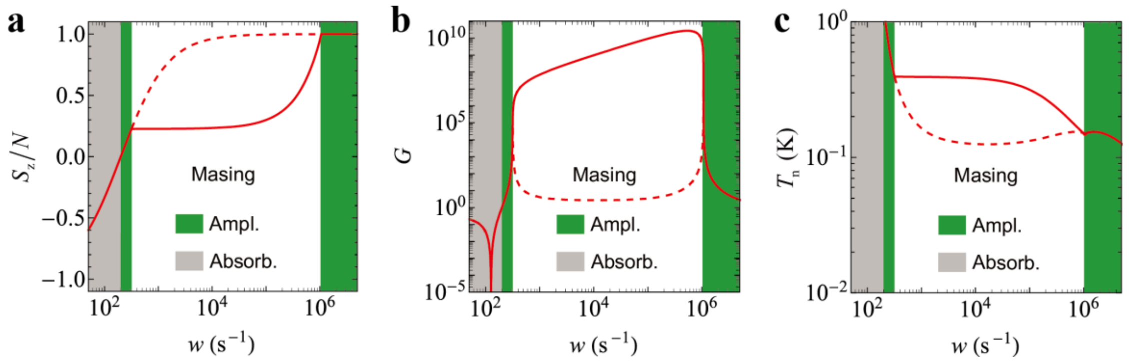

Supplementary Figure 1: Pump rate dependence of diamond microwave amplifier. (**a**) The spin polarization, (**b**) the power gain, and (**c**) the noise temperature as functions of the pump rate w for a weak resonant input signal P_{in}=1 fW and a fixed cavity Q factor Q=5×10^4. The absorbing (for w<γ_{eg}=200 s^{-1}), amplifying (for 200 s^{-1}<w<316 s^{-1}, w>1.07×10^6 s^{-1}), and masing regions (for 316 s^{-1}<w<1.07×10^6 s^{-1}) are marked as grey, green and white (see Supplementary Note 1 for pump thresholds). The solid lines are stable (masing) solutions and the dashed lines are the unstable (microwave amplifying) solutions in the masing region. The parameters are the same as in Fig. 2, i.e., $\omega_{in}/2\pi=\omega_c/2\pi=\omega_S/2\pi$=3 GHz, $g/2\pi$=0.02 Hz, T_2^*=0.4 μs, N=1.32×10^{14}, and γ_{eg}=200 s^{-1} at room-temperature (T=300 K).

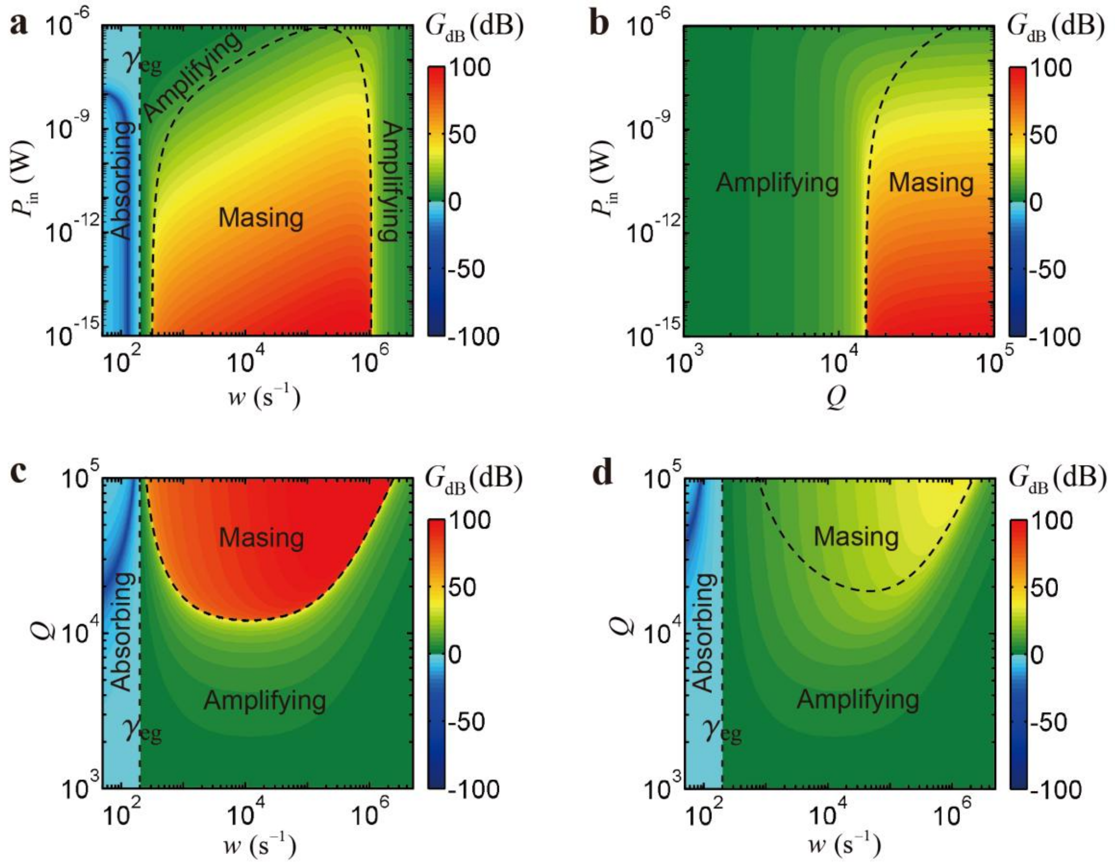

Supplementary Figure 2: Power gain of diamond microwave amplifier. The power gain in decibel as functions of pump rate, cavity loss, and resonant input power for (**a**) fixed cavity Q factor Q=5×10^4, (**b**) fixed pump rate w=10^5 s^{-1}, (**c**) fixed input power P_{in}=1 fW, and (**d**) fixed input power P_{in}=10 nW. The dashed black curve implies the masing threshold. The parameters are the same as in Fig. 2, i.e., $\omega_{in}/2\pi=\omega_c/2\pi=\omega_S/2\pi$=3 GHz, $g/2\pi$=0.02 Hz, T_2^*=0.4 μs, N=1.32×10^{14}, and γ_{eg}=200 s^{-1} at room-temperature (T=300 K).

Supplementary Note 1. Details of the diamond maser system

NV centre concentration. We consider a single crystal bulk diamond sample the same as used in Ref. [1]. The size of the diamond V_{NV}=3×3×0.5 mm^3. The P1 centre (single substitutional nitrogen centre) concentration is about 20 ppm (1 ppm=1.76×10^{17} cm^{-3}), and the NV centre concentration is about 2 ppm (corresponding to a 10% N-to-NV$^-$ conversion efficiency [1, 2]). For future improvement of the maser performance, we note that a conversion efficiency of up to 30% may be possible [3].

The number of active NV centre spins. The number of NV centre spins resonantly coupled to the cavity photons (N) is estimated as follows. Considering four orientations of the NV centres and the hyperfine interaction with the nitrogen nuclear spin (which splits the transition into three resonances, with only one resonant with the cavity mode), the number of NV centres effectively interacting with the cavity mode is estimated to be $N = \rho_{NV} V_{NV}/12 = 1.32 \times 10^{14}$. We note that for diamond fabricated with preferential alignment of NV centres in single-crystalline [4-6], the number of coupled NV centres is $N = \rho_{NV} V_{NV}/3$, and there will be 4 times more NV centres coupled to the cavity mode, which would lead to a 4 times reduction on the threshold cavity Q factor required for masing (Q_C is inversely proportional to N, see equation (5) in main text).

Spin dephasing time. The dephasing of an ensemble of NV centre spins is mainly caused by dipolar interaction with the P1 centre electron spins, the hyperfine interaction with ^{13}C nuclear spins, and the zero-field splitting (ZFS) fluctuations. The dipolar interaction with the P1 centre electron spins contributes a dephasing rate ~10^6 s^{-1} for the P1 concentration considered [7-10], interaction with the ^{13}C nuclear spins of natural abundance (1.1%) contributes ~10^6 s^{-1} [11, 12], and the zero-field splitting fluctuation induces a dephasing rate ~10^6 s^{-1} [1, 13]. Summing up these contributions, we estimate the total dephasing time T_2^*=0.4 μs for the ensemble of NV centres, which is close to the value measured in Ref. [1] in a diamond with the same parameters.

Spin-lattice relaxation time. The NV centre spin-lattice relaxation is temperature dependent. We adopt γ_{eg}=200 s^{-1} (T_1=5 ms) at room temperature T=300 K as taken from Refs. 14-16.

Microwave cavity. We consider a sapphire dielectric microwave resonator loaded in a coaxial cylindrical cavity, with a $TE_{01\delta}$ mode of frequency $\omega_c/2\pi$=3 GHz. The sapphire, for its low loss tangent (10^{-5}) at room temperature [17] and large dielectric permittivity (the relative permittivity $\varepsilon_r\approx10$ [18]), is an ideal material for room-temperature microwave resonators with high-Q ($Q>10^5$) and reduced mode volume [19]. The prime $TE_{01\delta}$ mode in the resonator has transverse electric field (solid red circle in Fig. 1a) and vertical magnetic field (dashed red circles in Fig. 1a) [20]. The magnetic field is azimuthally symmetric, and the magnetic loop on the side wall couples to the magnetic field. The electric field is quite strong in the equatorial plane of the resonator, except near the resonator centre. Consequently, removing the central part of the resonator will not disturb the field or the resonant frequency. A tubular resonator with 1/4 radius removed from the central still behaves similarly to a complete cylindrical dielectric resonator [21]. The diamond sample is placed in the centre of the sapphire resonator, with the NV axis oriented along the external magnetic field, which is applied perpendicular to the cavity axis. A mobile Halbach magnets array can provide a highly homogeneous 2,100 Gauss external magnetic field with inhomogeneity <0.01 Gauss across the diamond size (5 mm) [22-24]. The 532 nm pump light is input from the cavity side wall. The specific parameters of a cavity with desired features can be chosen such that the sapphire crystal is 16 mm in height (h), 15 mm in radius (r), which is loaded in the centre of a 40 mm height (H), 40 mm radius (R) coaxial cylindrical cavity, placed inside a Halbach magnet ring array with a 50 (80) mm inner (outer) radius [22, 23]. The exact resonant frequency of the $TE_{01\delta}$ mode in the cavity system, estimated from the parallel-plates model [25, 26], satisfies

$$\tan\left(\frac{h}{2}\sqrt{k_0^2\varepsilon_r-\frac{x_{01}^2}{r^2}}\right)\tanh\left(\frac{H-h}{2}\sqrt{\frac{x_{01}^2}{r^2}-k_0^2}\right)=\sqrt{\frac{x_{01}^2/r^2-k_0^2}{k_0^2\varepsilon_r-x_{01}^2/r^2}},\tag{1}$$

where $k_0=\omega_c/c$ is the wave number in free space, and $x_{01}\approx2.405$ is the first root of the Bessel function of the first kind, i.e., $J_0(x_{01})=0$.

Spin-photon coupling. The spin-photon coupling is $\frac{g}{2\pi}=\frac{\gamma_{NV}}{2\pi}\sqrt{\frac{\mu_0\hbar\omega_c}{2V_{eff}}}$, where V_{eff} is the effective cavity mode volume, and μ_0 is the vacuum permeability, $\hbar$ is the

Planck constant [27-30]. For a mode volume $V_{\text{eff}} \approx 3\,\text{cm}^3$ [20, 27], the coupling is $g/2\pi \approx 0.02$ Hz .

Thermal fluctuation of the cavity. The temperature variation leads to cavity mode frequency fluctuation via thermal expansion and dielectric constant variation. By equation (1), small variations in the dimensions of the dielectric resonator $r \to r+\Delta r$, $h \to h+\Delta h$ and in the permittivity $\varepsilon_{\text{r}} \to \varepsilon_{\text{r}}+\Delta\varepsilon_{\text{r}}$ lead to a small frequency shift $\Delta\omega_{\text{c}}$ of the $\text{TE}_{01\delta}$ mode, determined by

$$A\frac{\Delta\omega_{\text{c}}}{\omega_{\text{c}}}+B\frac{\Delta r}{r}+C\frac{\Delta h}{h}+D\frac{\Delta\varepsilon_{\text{r}}}{\varepsilon_{\text{r}}}=0, \tag{2}$$

with

$$A=\left(\frac{x_{01}}{r}\right)^2\frac{k_0^2(\varepsilon_{\text{r}}-1)}{\sqrt{\left(\frac{x_{01}}{r}\right)^2-k_0^2}}+k_0^2\varepsilon_{\text{r}}\frac{h}{2}\left[\frac{k_0^2\varepsilon_{\text{r}}-\left(\frac{x_{01}}{r}\right)^2}{\coth\zeta}+\frac{\left(\frac{x_{01}}{r}\right)^2-k_0^2}{\tanh\zeta}\right], \tag{3}$$

$$B=\left(\frac{x_{01}}{r}\right)^2\left\{\frac{k_0^2(\varepsilon_{\text{r}}-1)}{\sqrt{\left(\frac{x_{01}}{r}\right)^2-k_0^2}}+\frac{h}{2}\left[\frac{k_0^2\varepsilon_{\text{r}}-\left(\frac{x_{01}}{r}\right)^2}{\coth\zeta}+\frac{\left(\frac{x_{01}}{r}\right)^2-k_0^2}{\tanh\zeta}\right]-\frac{(H-h)\left[k_0^2\varepsilon_{\text{r}}-\left(\frac{x_{01}}{r}\right)^2\right]}{\sinh(2\zeta)}\right\}, \tag{4}$$

$$C=\left[k_0^2\varepsilon_{\text{r}}-\left(\frac{x_{01}}{r}\right)^2\right]\left\{\frac{h}{2}\left[\frac{k_0^2\varepsilon_{\text{r}}-\left(\frac{x_{01}}{r}\right)^2}{\coth\zeta}+\frac{\left(\frac{x_{01}}{r}\right)^2-k_0^2}{\tanh\zeta}\right]-\frac{h\left[\left(\frac{x_{01}}{r}\right)^2-k_0^2\right]}{\sinh(2\zeta)}\right\}, \tag{5}$$

$$D=\frac{1}{2}k_0^2\varepsilon_{\text{r}}\left\{\sqrt{\left(\frac{x_{01}}{r}\right)^2-k_0^2}+\frac{h}{2}\left[\frac{k_0^2\varepsilon_{\text{r}}-\left(\frac{x_{01}}{r}\right)^2}{\coth\zeta}+\frac{\left(\frac{x_{01}}{r}\right)^2-k_0^2}{\tanh\zeta}\right]\right\}, \tag{6}$$

where $\zeta=(H-h)\sqrt{(x_{01}/r)^2-k_0^2}\big/2$. The sapphire is an anisotropic material with properties such as hardness and thermal expansion different along different directions. For example, the thermal expansion coefficients of sapphire are different in directions perpendicular and parallel to the c-axis ($\alpha_{\perp}=5\times10^{-6}\ \text{K}^{-1}$ and $\alpha_{\parallel}=6.6\times10^{-6}\ \text{K}^{-1}$) [31],

i.e., $\Delta r/r = \alpha_{\perp}\Delta T$, and $\Delta h/h = \alpha_{\parallel}\Delta T$.

We approximate the sapphire as isotropic when considering the thermal expansion and dielectric constant variation, with the thermal expansion coefficient $\Delta r/r = \Delta h/h = \alpha\Delta T$ and the permittivity temperature coefficient $\Delta\varepsilon_{\mathrm{r}}/\varepsilon_{\mathrm{r}} = \beta\Delta T$. The mode frequency depends on the temperature via $\frac{\Delta\omega_{\mathrm{c}}}{\omega_{\mathrm{c}}} = -\left(\frac{B+C}{A}\alpha + \frac{D}{A}\beta\right)\Delta T$, the mode frequency fluctuation is

$$\Delta\omega_{\mathrm{c}} \approx -(\alpha + \beta/2)\omega_{\mathrm{c}}\Delta T, \tag{7}$$

where $\alpha = 5\times10^{-6}\ \mathrm{K}^{-1}$, $\beta = 10^{-4}\ \mathrm{K}^{-1}$ [18, 32], and $(2\pi)^{-1}\Delta\omega_{\mathrm{c}}/\Delta T$ is about $165\ \mathrm{kHz\ K}^{-1}$. The temperature fluctuation leads to additional cavity linewidth. Taking into account the cavity frequency fluctuation due to temperature instability, we introduce a reduced effective cavity Q factor Q^* via $\omega_{\mathrm{c}}/Q^* = \omega_{\mathrm{c}}/Q + \Delta\omega_{\mathrm{c}}$.

Thermal fluctuation of the NV centre spin. The zero-field splitting (D) of NV centre spin is also temperature dependent, with $\Delta D/\Delta T = -74\ \mathrm{kHz\ K}^{-1}$ [33]. Thus, the temperature fluctuation also leads to extra broadening of the spin transition frequency $\Delta\omega_{\mathrm{S}}/2\pi = (\Delta D/\Delta T)\cdot\Delta T$, which adds to $2/T_2^*$.

Optical pump process. The NV centre electronic spins are optically pumped into the m_{S}=0 ground state (Fig. 1c). A 532 nm green light excites the NV centre triplet ground state $^3A_2$ to vibronic excited states, which decay to the triplet excited state ^3E. The m_{S}=0 excited state almost fully decays to the ground state via spin-conserving photon emission. However, the m_{S}=±1 excited state can decay to the ground state either by spin-conserving photon emission or by spin non-conserving relaxation via the intersystem crossing (ISC), with similar rates [34-38]. From the singlet state $^1A_1$, the spin quickly decays to the metastable ^1E state at a rate about 1 ns^{-1} [34], and then relaxes back to the three ground states at roughly equal rates of 1 $\mu\mathrm{s}^{-1}$ [35-37]. Under continuous optical pumping, the m_{S}=−1 ground state ($|g\rangle$) is pumped to the m_{S}=0 ground state ($|e\rangle$) with a success probability of about 1/4 in each excitation event. Considering effective pump into $|e\rangle$ state at a rate w, the excitation rate is about $4w$ [37, 38]. The photon emission processes to the m_{S}=−1 and m_{S}=0 ground states have rates $2w$ and $4w$, respectively. All these pump and decay processes destroy the NV centre spin ensemble

coherence. Therefore the decay of the spin collective mode due to the optical pump has a rate about 16w. The total spin collective mode decay rate, including the contributions from the optical pump, the spin dephasing, and the spin-lattice relaxation, is thus obtained as $\kappa_S = qw + 2/T_2^* + \gamma_{eg}$, with $q \approx 16$.

Pump power. The absorption cross section of the NV centre for 532 nm light is $\sigma \approx 3.1\times10^{-17}$ cm^2 [39, 40]. The pump power is $P_{pump} = \hbar\omega_p(S/\sigma)(4w)$, where $\hbar\omega_p = 3.74\times10^{-19}$ J is the 532 nm photon energy, a light spot of radius 3 mm with illuminating area S=9π mm^2 fully covers the 3×3 mm^2 diamond sample. The absorbed power by the NV centres is $\hbar\omega_p(12N)(4w)$, only about 17% of the pump power.

Pump thresholds. The threshold pump rate for population inversion is $w = \gamma_{eg} = 200$ s^{-1}, below this threshold, the emitted photons are absorbed. Above this threshold, the population is inverted and there is net photons emission into the cavity. The maser will amplify the resonant input signal (Fig. 3 inserts and Supplementary Fig. 1). The threshold pump power for population inversion (microwave amplifying) determined from $w = \gamma_{eg} = 200$ s^{-1} is estimated to be 2.7 W.

The threshold pump rate for masing is when pump overcomes the cavity loss, which is obtained from the masing condition (equation (4) of the main text). Note that the total decay rate of collective NV centres spin mode is $\kappa_S = qw + \gamma_{eg} + 2/T_2^* \approx 2/T_2^*$ at low pump rate $w \sim \gamma_{eg}$. Thus, the threshold pump rate for masing is approximately $w_{th} \approx \gamma_{eg}\dfrac{1+\kappa_c/(2T_2^* g^2 N)}{1-\kappa_c/(2T_2^* g^2 N)}$, depending on the cavity decay rate. For the maser system with cavity Q factor Q=5×10^4, the threshold pump rate for masing is $w_{th} \approx 1.58\gamma_{eg} = 316$ s^{-1}, corresponding to a masing threshold pump power about 4.3 W. Note that for a good quality cavity, w_{th} will be close to but larger than γ_{eg} (e.g., $\gamma_{eg} < w_{th} < 1.11\gamma_{eg}$ for Q>2×10^5, see the masing boundary in Fig. 2, Fig. 3, and Supplementary Fig. 2c).

Furthermore, there exists an over-pump threshold for masing (w_{max}) at very large pump rate (see Fig. 3 inserts and Supplementary Fig. 1). Above this threshold, the

cavity field builds spin-spin correlation slower than the pump destroys it, thus masing will shut down. Note that the total decay rate of collective NV centres spin mode is $\kappa_S = qw + \gamma_{eg} + 2/T_2^* \approx qw + 2/T_2^*$ at high pump rate $w >> \gamma_{eg}$. From the masing condition (equation (4) of the main text), the over-pump threshold for masing is approximately $w_{max} \approx (4g^2N/\kappa_c - 2/T_2^*)/q$, depending on the cavity decay rate. Equivalently, masing shuts down when the spin collective mode decay rate κ_S reaches the maximal collective emission rate of photons $4Ng^2/\kappa_c$, for the pump would fully polarize the spins, making the spin-spin correlation vanish.

Masing threshold. The masing threshold sets a restriction on the cavity *Q* factor, i.e., $Q > \omega_c\kappa_S/\left(4Ng^2\right)$ or equivalently $\kappa_c < 4g^2N/\kappa_S$. Although a larger number of NV centres *N* can reduce the threshold cavity *Q* factor, increasing the NV centre density and hence the P1 centre density would reduce the spin dephasing time T_2^* (which is inversely proportional to the P1 centre density, $\rho_{P1}T_2^* \approx 8.8\times10^{17}\ \mathrm{m}^{-3}\ \mathrm{s}$ [7, 41]). Taking the NV centre spin relaxation rate $\kappa_S > 2/T_2^*$, we have $N/\kappa_S < \rho_{NV}V_{NV}T_2^*/24$. Substituting the spin-photon coupling *g* and N/κ_S into the masing threshold inequality, we get $Q\cdot\left(V_{NV}/V_{eff}\right)\cdot\left(\gamma_{NV}^2\mu_0\hbar/12\right)\cdot\left(\rho_{NV}T_2^*\right) > 1$. Assuming a 10% N-to-NV conversion efficiency, we obtain $Q > Q_C$ with threshold cavity *Q* factor

$$Q_C \approx 30V_{eff}/V_{NV.} \tag{8}$$

Increasing the filling factor V_{NV}/V_{eff} will reduce the threshold cavity *Q* factor. For a 4.5 mm^3 diamond and V_{eff} confined below 15 cm^3, the threshold cavity *Q* factor is below 10^5. Moreover, using larger size diamond can also reduce the threshold cavity *Q* factor but requires higher pump power.

Supplementary Note 2. Langevin equations and steady-state solution

The quantum Langevin equations read [42]

$$\frac{\mathrm{d}\hat{N}_e}{\mathrm{d}t} = +w\hat{N}_g - \gamma_{eg}\hat{N}_e + ig\left(\hat{a}^\dagger\hat{S}_- - \hat{S}_+\hat{a}\right) + \hat{F}_e, \tag{9}$$

$$\frac{\mathrm{d}\hat{N}_{\mathrm{g}}}{\mathrm{d}t} = -w\hat{N}_{\mathrm{g}} + \gamma_{\mathrm{eg}}\hat{N}_{\mathrm{e}} - ig\left(\hat{a}^{\dagger}\hat{S}_{-} - \hat{S}_{+}\hat{a}\right) + \hat{F}_{\mathrm{g}}, \tag{10}$$

$$\frac{\mathrm{d}\hat{S}_{-}}{\mathrm{d}t} = -i\omega_{\mathrm{S}}\hat{S}_{-} - \frac{\kappa_{\mathrm{S}}}{2}\hat{S}_{-} + ig\hat{S}_{\mathrm{z}}\hat{a} + \hat{F}_{\mathrm{S}}, \tag{11}$$

$$\frac{\mathrm{d}\hat{a}}{\mathrm{d}t} = -i\omega_{\mathrm{c}}\hat{a} - \frac{\kappa_{\mathrm{c}}}{2}\hat{a} - ig\hat{S}_{-} + \hat{F}_{\mathrm{c}}, \tag{12}$$

where $\hat{N}_{\mathrm{e}} - \hat{N}_{\mathrm{g}} = \hat{S}_{\mathrm{z}}$, $\hat{N}_{\mathrm{e}} + \hat{N}_{\mathrm{g}} = \hat{N}$, the noise operator $\hat{F}_{\mathrm{e/g}}$ is the population fluctuation in the spin state $\left|\mathrm{e/g}\right\rangle$, $\hat{F}_{\mathrm{e}} + \hat{F}_{\mathrm{g}}$ is the fluctuation of the total NV centre electron spin number $\hat{N}$ due to population in the m_{S}=+1 state and other intermediate states, and $\hat{F}_{\mathrm{S/c}}$ is the spin collective mode/photon noise. We take N as the steady-state average value of $\hat{N}$.

The mean-field theory is well justified when masing occurs, since the fluctuations are much smaller than the expectation values of the operators in equations (9)-(12). We write the operators as the sum of their expectation values and small fluctuations, i.e., $\hat{N}_{\mathrm{e/g}} = N_{\mathrm{e/g}} + \delta\hat{N}_{\mathrm{e/g}}$, $\hat{S}_{\pm} = S_{\pm}e^{\pm i\omega t} + \delta\hat{S}_{\pm}$, $\hat{a} = ae^{-i\omega t} + \delta\hat{a}$, where ω is the steady-state frequency of the maser. The steady-state mean-field equations are

$$0 = wN_{\mathrm{g}} - \gamma_{\mathrm{eg}}N_{\mathrm{e}} + ig(a^{*}S_{-} - S_{+}a), \tag{13}$$

$$0 = i(\omega - \omega_{\mathrm{S}})S_{-} - \frac{\kappa_{\mathrm{S}}}{2}S_{-} + igS_{\mathrm{z}}a, \tag{14}$$

$$0 = i(\omega - \omega_{\mathrm{c}})a - \frac{\kappa_{\mathrm{c}}}{2}a - igS_{-}, \tag{15}$$

From equations (13)-(15), we obtain

$$S_{-} = \frac{-igS_{\mathrm{z}}a}{i(\omega-\omega_{\mathrm{S}}) - \frac{\kappa_{\mathrm{S}}}{2}}, \quad a = \frac{igS_{-}}{i(\omega-\omega_{\mathrm{c}}) - \frac{\kappa_{\mathrm{c}}}{2}}, \tag{16}$$

which gives the spin polarization (population inversion)

$$S_{\mathrm{z}} = \frac{\left[\kappa_{\mathrm{c}} - 2i(\omega-\omega_{\mathrm{c}})\right]\left[\kappa_{\mathrm{S}} - 2i(\omega-\omega_{\mathrm{S}})\right]}{4g^{2}}. \tag{17}$$

The inversion S_{z} being real requires $(\omega-\omega_{\mathrm{c}})\kappa_{\mathrm{S}} + (\omega-\omega_{\mathrm{S}})\kappa_{\mathrm{c}} = 0$, which determines the maser frequency $\omega = \frac{\kappa_{\mathrm{c}}\omega_{\mathrm{S}} + \kappa_{\mathrm{S}}\omega_{\mathrm{c}}}{\kappa_{\mathrm{c}} + \kappa_{\mathrm{S}}}$. Inserting ω into equation (17), we get

$$S_z = \frac{\kappa_S \kappa_c}{4g^2}\left(1+\delta_{cs}^2\right), \tag{18}$$

where $\delta_{cs} = 2(\omega_c - \omega_S)/(\kappa_c + \kappa_S)$ is the frequency mismatch between the spin transition and cavity [43]. At resonance $\omega_c = \omega_S = \omega,$ the solutions are $\kappa_S \kappa_c = 4g^2 S_z,$ $\kappa_c a = -2igS_-,$ and the spin collective mode and photon numbers satisfy $\kappa_c n_c = \kappa_S \left(S_+ S_- / S_z\right) = \kappa_S n_S$.

To calculate the spin-spin correlation, we use

$$\frac{\mathrm{d}\left\langle \hat{N}_e \right\rangle}{\mathrm{d}t} = w\left\langle \hat{N}_g \right\rangle - \gamma_{eg}\left\langle \hat{N}_e \right\rangle + ig\left(\left\langle \hat{a}^\dagger \hat{S}_- \right\rangle - \left\langle \hat{S}_+ \hat{a} \right\rangle\right), \tag{19}$$

$$\frac{\mathrm{d}\left\langle \hat{N}_g \right\rangle}{\mathrm{d}t} = -w\left\langle \hat{N}_g \right\rangle + \gamma_{eg}\left\langle \hat{N}_e \right\rangle - ig\left(\left\langle \hat{a}^\dagger \hat{S}_- \right\rangle - \left\langle \hat{S}_+ \hat{a} \right\rangle\right), \tag{20}$$

$$\frac{\mathrm{d}\left\langle \hat{a}^\dagger \hat{S}_- \right\rangle}{\mathrm{d}t} = -\frac{\kappa_S + \kappa_c}{2}\left\langle \hat{a}^\dagger \hat{S}_- \right\rangle + ig\left[(1-\frac{1}{N})\left\langle \hat{S}_+ \hat{S}_- \right\rangle + \left\langle \hat{N}_e \right\rangle + \left\langle \hat{a}^\dagger \hat{a} \right\rangle\left\langle \hat{S}_z \right\rangle\right], \tag{21}$$

$$\frac{\mathrm{d}\left\langle \hat{S}_+ \hat{S}_- \right\rangle}{\mathrm{d}t} = -\kappa_S\left\langle \hat{S}_+ \hat{S}_- \right\rangle - ig\left\langle \hat{S}_z \right\rangle\left(\left\langle \hat{a}^\dagger \hat{S}_- \right\rangle - \left\langle \hat{S}_+ \hat{a} \right\rangle\right), \tag{22}$$

$$\frac{\mathrm{d}\left\langle \hat{a}^\dagger \hat{a} \right\rangle}{\mathrm{d}t} = -\kappa_c\left\langle \hat{a}^\dagger \hat{a} \right\rangle - ig\left(\left\langle \hat{a}^\dagger \hat{S}_- \right\rangle - \left\langle \hat{S}_+ \hat{a} \right\rangle\right) + \kappa_c n_{th}, \tag{23}$$

where symbol $\langle \cdots \rangle$ denotes the expectation values of the operators. We have used the approximations $\left\langle \hat{a}^\dagger \hat{a} \hat{S}_z \right\rangle \approx \left\langle \hat{a}^\dagger \hat{a} \right\rangle\left\langle \hat{S}_z \right\rangle$, $\left\langle \hat{a}^\dagger \hat{S}_z \hat{S}_- \right\rangle \approx \left\langle \hat{S}_z \right\rangle\left\langle \hat{a}^\dagger \hat{S}_- \right\rangle$, and $\left\langle \hat{S}_+ \hat{S}_z \hat{a} \right\rangle \approx \left\langle \hat{S}_z \right\rangle\left\langle \hat{S}_+ \hat{a} \right\rangle$, dropping the higher order correlations [44, 45]. The steady-state expectation values are calculated by setting $\mathrm{d}\left\langle \hat{N}_{e/g} \right\rangle / \mathrm{d}t = \mathrm{d}\left\langle \hat{a}^\dagger \hat{S}_- \right\rangle / \mathrm{d}t = \mathrm{d}\left\langle \hat{S}_+ \hat{S}_- \right\rangle / \mathrm{d}t = \mathrm{d}\left\langle \hat{a}^\dagger \hat{a} \right\rangle / \mathrm{d}t = 0$ in equations (19)-(23), which leads to

$$0 = wN_g - \gamma_{eg} N_e + ig\left(\left\langle \hat{a}^\dagger \hat{S}_- \right\rangle - \left\langle \hat{S}_+ \hat{a} \right\rangle\right), \tag{24}$$

$$0 = -\frac{\kappa_S + \kappa_c}{2}\left\langle \hat{a}^\dagger \hat{S}_- \right\rangle + ig\left[(1-\frac{1}{N})\left\langle \hat{S}_+ \hat{S}_- \right\rangle + N_e + \left\langle \hat{a}^\dagger \hat{a} \right\rangle S_z\right], \tag{25}$$

$$0 = -\kappa_S\left\langle \hat{S}_+ \hat{S}_- \right\rangle - igS_z\left(\left\langle \hat{a}^\dagger \hat{S}_- \right\rangle - \left\langle \hat{S}_+ \hat{a} \right\rangle\right), \tag{26}$$

$$0 = -\kappa_c\left\langle \hat{a}^\dagger \hat{a} \right\rangle - ig\left(\left\langle \hat{a}^\dagger \hat{S}_- \right\rangle - \left\langle \hat{S}_+ \hat{a} \right\rangle\right) + \kappa_c n_{th}. \tag{27}$$

The spin-spin correlation $\left\langle \hat{S}_+\hat{S}_- \right\rangle$, the populations of the two spin states $N_{\mathrm{e/g}}$, the spin polarization S_{z}, and the photon number n_{c} are then obtained from equations (24)-(27). The incoherent thermal photons are far less than the stimulated emission photons and mainly affect the linewidth of the microwave field. Above the threshold for population inversion ($w > \gamma_{\mathrm{eg}}$), we can safely neglect n_{th} in equations (24)-(27). Solving equations (24)-(27) yields a quadratic equation about S_{z}, that is,

$$S_{\mathrm{z}}^2 - \left[\frac{w-\gamma_{\mathrm{eg}}}{w+\gamma_{\mathrm{eg}}} N + \frac{\frac{\kappa_{\mathrm{S}}+\kappa_{\mathrm{c}}}{4g^2} + \frac{1}{w+\gamma_{\mathrm{eg}}}}{(1-N^{-1})\frac{1}{\kappa_{\mathrm{S}}} + \frac{1}{\kappa_{\mathrm{c}}}} \right] S_{\mathrm{z}} + \frac{w-\gamma_{\mathrm{eg}}}{w+\gamma_{\mathrm{eg}}} N \frac{\frac{\kappa_{\mathrm{S}}+\kappa_{\mathrm{c}}}{4g^2} - \frac{1}{w-\gamma_{\mathrm{eg}}}}{(1-N^{-1})\frac{1}{\kappa_{\mathrm{S}}} + \frac{1}{\kappa_{\mathrm{c}}}} = 0. \quad (28)$$

Since $\frac{\kappa_{\mathrm{S}}+\kappa_{\mathrm{c}}}{4g^2} >> \frac{1}{w\pm\gamma_{\mathrm{eg}}}$ and $N >> 1$, we have $\frac{\kappa_{\mathrm{S}}+\kappa_{\mathrm{c}}}{4g^2} \pm \frac{1}{w\pm\gamma_{\mathrm{eg}}} \approx \frac{\kappa_{\mathrm{S}}+\kappa_{\mathrm{c}}}{4g^2}$ and $(1-N^{-1}) \approx 1$. Equation (28) is further reduced to

$$S_{\mathrm{z}}^2 - \left(\frac{w-\gamma_{\mathrm{eg}}}{w+\gamma_{\mathrm{eg}}} N + \frac{\kappa_{\mathrm{S}}\kappa_{\mathrm{c}}}{4g^2} \right) S_{\mathrm{z}} + \frac{w-\gamma_{\mathrm{eg}}}{w+\gamma_{\mathrm{eg}}} N \times \frac{\kappa_{\mathrm{S}}\kappa_{\mathrm{c}}}{4g^2} = 0. \quad (29)$$

The population inversion in the masing region is given by the stable solution

$$S_{\mathrm{z}} = \frac{\kappa_{\mathrm{S}}\kappa_{\mathrm{c}}}{4g^2}. \quad (30)$$

And in the amplifying region, the stable solution is $S_{\mathrm{z}} = \frac{w-\gamma_{\mathrm{eg}}}{w+\gamma_{\mathrm{eg}}} N$ (details of amplifying is discussed in Supplementary Note 5).

Supplementary Note 3. Spin-spin correlation

The spin-spin correlation can be determined from equations (24)-(27), as

$$\left\langle \hat{S}_+\hat{S}_- \right\rangle = \frac{w}{2\kappa_{\mathrm{S}}} S_{\mathrm{z}} \left[(1-\frac{\gamma_{\mathrm{eg}}}{w})N - (1+\frac{\gamma_{\mathrm{eg}}}{w})S_{\mathrm{z}} \right]. \quad (31)$$

It reaches the maximum value

$$\left\langle \hat{S}_+\hat{S}_- \right\rangle = \frac{N^2}{8q} \left(1 - \frac{\kappa_{\mathrm{c}}}{2Ng^2T_2^*} \right)^2, \quad (32)$$

at pump rate $w = \left(2Ng^2/\kappa_c - 1/T_2^*\right)/q$. The maximum spin-spin correlation is proportional to N^2 provided that $2Ng^2T_2^* >> \kappa_c$, which is satisfied when the maser operates well above the threshold. Maximum spin-spin correlation is reached when the pump rate $w = \left(2Ng^2/\kappa_c - 1/T_2^*\right)/q$, where the spin ensemble is at half inversion ($S_z = N/2$). The incoherent population is mainly the thermal photons ($n_{incoh} \approx n_{th}$) at room temperature ($n_{th} >> N_e/S_z = 3/2$). Thus, the diffusion coefficient in equation (44) becomes $\gamma_{ST} \approx n_{th}\,\kappa_c/\left(2n_c\right)$, and the minimal diffusion coefficient and maximal coherence time are $\gamma_{ST} \approx qn_{th}\,\kappa_c^3/\left(N^2g^2\right)$ and $T_{coh} \approx 2N^2g^2/\left(qn_{th}\kappa_c^3\right)$, respectively (see Supplementary Note 4 for quantum diffusion γ_{ST} and coherence time T_{coh}).

Supplementary Note 4. Quantum diffusion and coherence time

The linearized quantum Langevin equations for the fluctuations are (second order terms such as $\delta\hat{a}^\dagger\delta\hat{S}_-$ dropped)

$$\frac{\mathrm{d}\delta\hat{N}_e}{\mathrm{d}t} = +w\delta\hat{N}_g - \gamma_{eg}\delta\hat{N}_e + ig(S_-\delta\hat{a}^\dagger - S_+\delta\hat{a}) + ig(a^*\delta\hat{S}_- - a\delta\hat{S}_+) + \hat{F}_e, \qquad (33)$$

$$\frac{\mathrm{d}\delta\hat{N}_g}{\mathrm{d}t} = -w\delta\hat{N}_g + \gamma_{eg}\delta\hat{N}_e - ig(S_-\delta\hat{a}^\dagger - S_+\delta\hat{a}) - ig(a^*\delta\hat{S}_- - a\delta\hat{S}_+) + \hat{F}_g, \qquad (34)$$

$$\frac{\mathrm{d}\delta\hat{S}_-}{\mathrm{d}t} = -\frac{\kappa_S}{2}\delta\hat{S}_- + igS_z\delta\hat{a} + iga(\delta\hat{N}_e - \delta\hat{N}_g) + \hat{F}_S, \qquad (35)$$

$$\frac{\mathrm{d}\delta\hat{a}}{\mathrm{d}t} = -\frac{\kappa_c}{2}\delta\hat{a} - ig\delta\hat{S}_- + \hat{F}_c. \qquad (36)$$

Here we have assumed the resonant condition $\omega_c = \omega_S$. By Fourier transform $\delta\hat{a}(\Omega) = (2\pi)^{-1/2}\int_{-\infty}^{+\infty} e^{i\Omega t}\delta\hat{a}(t)\mathrm{d}t$ for $\delta\hat{a}(t)$ and other fluctuation operators, we obtain the noise operators in the frequency domain. Without loss of generality, we set the intracavity field a as a real number. Under the resonant condition, the spin collective mode amplitude S_- is purely imaginary.

The quantum Langevin equations in frequency domain yield

$$-i\Omega\delta\hat{S}_{-}(\Omega)=-\frac{\kappa_{\mathrm{S}}}{2}\delta\hat{S}_{-}(\Omega)+iga[\delta\hat{N}_{\mathrm{e}}(\Omega)-\delta\hat{N}_{\mathrm{g}}(\Omega)]+igS_{z}\delta\hat{a}(\Omega)+\hat{F}_{\mathrm{S}}(\Omega), \quad (37)$$

$$-i\Omega\delta\hat{S}_{+}(\Omega)=-\frac{\kappa_{\mathrm{S}}}{2}\delta\hat{S}_{+}(\Omega)-iga^{*}[\delta\hat{N}_{\mathrm{e}}(\Omega)-\delta\hat{N}_{\mathrm{g}}(\Omega)]-igS_{z}\delta\hat{a}^{\dagger}(\Omega)+\hat{F}_{\mathrm{S}}^{\dagger}(\Omega), \quad (38)$$

$$-i\Omega\delta\hat{a}(\Omega)=-\frac{\kappa_{\mathrm{c}}}{2}\delta\hat{a}(\Omega)-ig\delta\hat{S}_{-}(\Omega)+\hat{F}_{\mathrm{c}}(\Omega), \quad (39)$$

$$-i\Omega\delta\hat{a}^{\dagger}(\Omega)=-\frac{\kappa_{\mathrm{c}}}{2}\delta\hat{a}^{\dagger}(\Omega)+ig\delta\hat{S}_{+}(\Omega)+\hat{F}_{\mathrm{c}}^{\dagger}(\Omega). \quad (40)$$

The phase fluctuation of spin collective modes is

$$\delta\hat{\phi}_{\mathrm{S}}=[\delta\hat{S}_{-}(\Omega)+\delta\hat{S}_{+}(\Omega)]=\frac{i(\frac{\kappa_{\mathrm{c}}}{2}-i\Omega)[\hat{F}_{\mathrm{S}}(\Omega)+\hat{F}_{\mathrm{S}}^{\dagger}(\Omega)]-\frac{\kappa_{\mathrm{c}}\kappa_{\mathrm{S}}}{4g}[\hat{F}_{\mathrm{c}}(\Omega)-\hat{F}_{\mathrm{c}}^{\dagger}(\Omega)]}{\Omega(\frac{\kappa_{\mathrm{c}}+\kappa_{\mathrm{S}}}{2}-i\Omega)}, \quad (41)$$

and that of photons is

$$\delta\hat{\phi}_{\mathrm{c}}(\Omega)=-i[\delta\hat{a}(\Omega)-\delta\hat{a}^{\dagger}(\Omega)]=\frac{(\frac{\kappa_{\mathrm{S}}}{2}-i\Omega)[\hat{F}_{\mathrm{c}}(\Omega)-\hat{F}_{\mathrm{c}}^{\dagger}(\Omega)]-ig[\hat{F}_{\mathrm{S}}(\Omega)+\hat{F}_{\mathrm{S}}^{\dagger}(\Omega)]}{\Omega(\frac{\kappa_{\mathrm{c}}+\kappa_{\mathrm{S}}}{2}-i\Omega)}. \quad (42)$$

With the correlation functions of the field noise operators, $\left\langle\hat{F}_{\mathrm{c}}(\Omega)\hat{F}_{\mathrm{c}}(\Omega')\right\rangle=0$, $\left\langle\hat{F}_{\mathrm{c}}^{\dagger}(\Omega)\hat{F}_{\mathrm{c}}(\Omega')\right\rangle=\kappa_{\mathrm{c}}n_{\mathrm{th}}\delta(\Omega+\Omega')$, and $\left\langle\hat{F}_{\mathrm{c}}(\Omega)\hat{F}_{\mathrm{c}}^{\dagger}(\Omega')\right\rangle=\kappa_{\mathrm{c}}(1+n_{\mathrm{th}})\delta(\Omega+\Omega')$, we obtain $[\hat{F}_{\mathrm{c}}(\Omega)-\hat{F}_{\mathrm{c}}^{\dagger}(\Omega)][\hat{F}_{\mathrm{c}}(\Omega')-\hat{F}_{\mathrm{c}}^{\dagger}(\Omega')]=-\kappa_{\mathrm{c}}(1+2n_{\mathrm{th}})\delta(\Omega+\Omega')$. Similarly, the noise operators for the spins satisfy $[\hat{F}_{\mathrm{S}}(\Omega)+\hat{F}_{\mathrm{S}}^{\dagger}(\Omega)][\hat{F}_{\mathrm{S}}(\Omega')+\hat{F}_{\mathrm{S}}^{\dagger}(\Omega')]=N\kappa_{\mathrm{S}}\delta(\Omega+\Omega')$.

The phase noise spectrum $S_{\mathrm{c}}(\Omega)=\left\langle\delta\hat{\phi}_{\mathrm{c}}(\Omega)\delta\hat{\phi}_{\mathrm{c}}(-\Omega)\right\rangle$ is calculated as

$$\frac{S_{\mathrm{c}}(\Omega)}{4n_{\mathrm{c}}}=\frac{\left(\frac{\kappa_{\mathrm{c}}+\kappa_{\mathrm{S}}}{2}\right)^{2}}{\Omega^{2}\left[\left(\frac{\kappa_{\mathrm{c}}+\kappa_{\mathrm{S}}}{2}\right)^{2}+\Omega^{2}\right]}\left[\frac{g^{2}N\kappa_{\mathrm{S}}+\left(\frac{\kappa_{\mathrm{S}}^{2}}{4}+\Omega^{2}\right)\kappa_{\mathrm{c}}(1+2n_{\mathrm{th}})}{4n_{\mathrm{c}}\left(\frac{\kappa_{\mathrm{c}}+\kappa_{\mathrm{S}}}{2}\right)^{2}}\right]. \quad (43)$$

The maser linewidth is determined by the phase noises at low frequencies, $\Omega<<\left(\kappa_{\mathrm{c}}+\kappa_{\mathrm{S}}\right)/2$. The linewidth is much less than the cavity and spin collective mode decay rates, so the time derivative of the maser phase fluctuation correlation function can be approximated as $\left\langle\frac{\mathrm{d}}{\mathrm{d}t}\delta\hat{\phi}_{\mathrm{c}}(t)\frac{\mathrm{d}}{\mathrm{d}t'}\delta\hat{\phi}_{\mathrm{c}}(t')\right\rangle=\frac{1}{2\pi}\int_{-\infty}^{+\infty}e^{-i\Omega(t-t')}\Omega^{2}S_{\mathrm{c}}(\Omega)\mathrm{d}\Omega$

$\approx \gamma_{\mathrm{ST}}\delta(t-t')$, where

$$\gamma_{\mathrm{ST}} = \left(\frac{N_{\mathrm{e}}}{S_{\mathrm{z}}} + n_{\mathrm{th}}\right)\frac{\kappa_{\mathrm{c}}}{2n_{\mathrm{c}}}\left(\frac{\kappa_{\mathrm{S}}}{\kappa_{\mathrm{c}}+\kappa_{\mathrm{S}}}\right)^2 = n_{\mathrm{incoh}} \cdot \frac{1}{n_{\mathrm{c}}+n_{\mathrm{S}}} \cdot \frac{1}{(\kappa_{\mathrm{S}}/2)^{-1}+(\kappa_{\mathrm{c}}/2)^{-1}}, \tag{44}$$

is the Schawlow-Townes diffusion coefficient, with incoherent spin collective mode and photon number $n_{\mathrm{incoh}} = N_{\mathrm{e}}/S_{\mathrm{z}} + n_{\mathrm{th}}$. For $t >> 2/(\kappa_{\mathrm{c}}+\kappa_{\mathrm{S}})$, the phase correlation is $\left\langle \delta\hat{\phi}_{\mathrm{c}}^2(t)\right\rangle = \int_0^t \mathrm{d}t' \int_0^t \mathrm{d}t'' \left\langle \delta\dot{\hat{\phi}}_{\mathrm{c}}(t')\delta\dot{\hat{\phi}}_{\mathrm{c}}(t'')\right\rangle = \gamma_{\mathrm{ST}} \cdot t$. Neglecting the amplitude fluctuation (which is negligible at steady-state) and assuming a Gaussian statistics for the phase fluctuation, the spectrum of the cavity photon field is

$$\begin{aligned} \left\langle \hat{a}^\dagger(\Omega)\hat{a}(-\Omega)\right\rangle &= \int_{-\infty}^{+\infty} \mathrm{d}t e^{i\Omega t} \left\langle \hat{a}^\dagger(t)\hat{a}(0)\right\rangle \\ &= n_{\mathrm{c}}\int_{-\infty}^{+\infty} \mathrm{d}t e^{i(\Omega-\omega_{\mathrm{c}})t} e^{-\left\langle \delta\hat{\phi}_{\mathrm{c}}^2(t)\right\rangle/2} = n_{\mathrm{c}} \frac{\gamma_{\mathrm{ST}}}{(\Omega-\omega_{\mathrm{c}})^2 + (\gamma_{\mathrm{ST}}/2)^2}. \end{aligned} \tag{45}$$

The photon field has a full-width-half-maximum linewidth $\gamma_{\mathrm{ST}}/2\pi$. Correspondingly, the coherence time of the maser is $T_{\mathrm{coh}} = 2/\gamma_{\mathrm{ST}}$.

Supplementary Note 5. Diamond microwave amplifier

With an input signal $s_{\mathrm{in}}e^{-i\omega_{\mathrm{in}}t}$, the steady-state Langevin equations are

$$0 = wN_{\mathrm{g}} - \gamma_{\mathrm{eg}}N_{\mathrm{e}} + ig(a^*S_- - S_+a), \tag{46}$$

$$0 = i(\omega_{\mathrm{in}} - \omega_{\mathrm{S}})S_- - \frac{\kappa_{\mathrm{S}}}{2}S_- + igS_{\mathrm{z}}a, \tag{47}$$

$$0 = i(\omega_{\mathrm{in}} - \omega_{\mathrm{c}})a - \frac{\kappa_{\mathrm{c}}}{2}a - igS_- + \sqrt{\kappa_{\mathrm{ex}}}s_{\mathrm{in}}, \tag{48}$$

$$s_{\mathrm{out}} = s_{\mathrm{in}} - \sqrt{\kappa_{\mathrm{ex}}}a. \tag{49}$$

With the input signal detuning denoted as $\delta_{\mathrm{S,c}} = \omega_{\mathrm{in}} - \omega_{\mathrm{S,c}}$, the inversion and output satisfy

$$\frac{w-\gamma_{\mathrm{eg}}}{w+\gamma_{\mathrm{eg}}} = \left\{1 + \frac{\frac{2\kappa_{\mathrm{ex}}}{\kappa_{\mathrm{c}}}\frac{4g^2}{\kappa_{\mathrm{S}}\kappa_{\mathrm{c}}}\frac{4|s_{\mathrm{in}}|^2}{w+\gamma_{\mathrm{eg}}}}{\left(\frac{4\delta_{\mathrm{S}}^2}{\kappa_{\mathrm{S}}^2}+1\right)\left(\frac{4\delta_{\mathrm{c}}^2}{\kappa_{\mathrm{c}}^2}+1\right) - 1 + 2\frac{4\delta_{\mathrm{S}}\delta_{\mathrm{c}}}{\kappa_{\mathrm{S}}\kappa_{\mathrm{c}}}\frac{4g^2}{\kappa_{\mathrm{S}}\kappa_{\mathrm{c}}}S_{\mathrm{z}} + \left(\frac{4g^2}{\kappa_{\mathrm{S}}\kappa_{\mathrm{c}}}S_{\mathrm{z}} - 1\right)^2}\right\}\frac{S_{\mathrm{z}}}{N}, \tag{50}$$

$$s_{\mathrm{out}} = \frac{\left(1-\frac{2\kappa_{\mathrm{ex}}}{\kappa_{\mathrm{c}}}\right) - \frac{4\delta_{\mathrm{S}}\delta_{\mathrm{c}}}{\kappa_{\mathrm{S}}\kappa_{\mathrm{c}}} - \frac{4g^2}{\kappa_{\mathrm{S}}\kappa_{\mathrm{c}}} S_{\mathrm{z}} - i\left[\left(1-\frac{2\kappa_{\mathrm{ex}}}{\kappa_{\mathrm{c}}}\right)\frac{2\delta_{\mathrm{S}}}{\kappa_{\mathrm{S}}} + \frac{2\delta_{\mathrm{c}}}{\kappa_{\mathrm{c}}}\right]}{1 - \frac{4\delta_{\mathrm{S}}\delta_{\mathrm{c}}}{\kappa_{\mathrm{S}}\kappa_{\mathrm{c}}} - \frac{4g^2}{\kappa_{\mathrm{S}}\kappa_{\mathrm{c}}} S_{\mathrm{z}} - i\left(\frac{2\delta_{\mathrm{S}}}{\kappa_{\mathrm{S}}} + \frac{2\delta_{\mathrm{c}}}{\kappa_{\mathrm{c}}}\right)} s_{\mathrm{in}}. \tag{51}$$

The power gain is $G = |s_{\mathrm{out}}|^2 / |s_{\mathrm{in}}|^2$. For detuning between the cavity, electron spin, and the input signal frequencies $\delta_{\mathrm{S,c}}/\kappa_{\mathrm{S,c}} \gtrsim 1$, the power gain would be significantly reduced to $G \sim O(1)$. However, large power gain is possible under the resonant input condition $\delta_{\mathrm{S,c}} = 0$.

In the following, we assume the resonant input condition. We also assume the cavity loss is mainly caused by the coupling to the input-output channel, i.e. $\kappa_{\mathrm{ex}} = \kappa_{\mathrm{c}}$. The inversion reduces to an exactly solvable cubic equation

$$\frac{w-\gamma_{\mathrm{eg}}}{w+\gamma_{\mathrm{eg}}} N = \left[1 + 2\frac{4g^2}{\kappa_{\mathrm{S}}\kappa_{\mathrm{c}}} \frac{4|s_{\mathrm{in}}|^2}{w+\gamma_{\mathrm{eg}}} \left(1 - \frac{4g^2}{\kappa_{\mathrm{S}}\kappa_{\mathrm{c}}} S_{\mathrm{z}}\right)^{-2}\right] S_{\mathrm{z}}. \tag{52}$$

and the power gain reduces to

$$G = \left(1 + \frac{4g^2}{\kappa_{\mathrm{S}}\kappa_{\mathrm{c}}} S_{\mathrm{z}}\right)^2 \Big/ \left(1 - \frac{4g^2}{\kappa_{\mathrm{S}}\kappa_{\mathrm{c}}} S_{\mathrm{z}}\right)^2. \tag{53}$$

In the amplifying region, we approximately obtain the population inversion for weak input signal $|s_{\mathrm{in}}|^2 << \frac{g^2(w+\gamma_{\mathrm{eg}})}{2\kappa_{\mathrm{S}}\kappa_{\mathrm{c}}}(\frac{w-\gamma_{\mathrm{eg}}}{w+\gamma_{\mathrm{eg}}} N - \frac{\kappa_{\mathrm{S}}\kappa_{\mathrm{c}}}{4g^2})^2$ as

$$S_{\mathrm{z}} = \frac{w-\gamma_{\mathrm{eg}}}{w+\gamma_{\mathrm{eg}}} N \left[1 - 2\frac{\kappa_{\mathrm{S}}\kappa_{\mathrm{c}}}{4g^2} \frac{4|s_{\mathrm{in}}|^2}{w+\gamma_{\mathrm{eg}}} \left(\frac{w-\gamma_{\mathrm{eg}}}{w+\gamma_{\mathrm{eg}}} N - \frac{\kappa_{\mathrm{S}}\kappa_{\mathrm{c}}}{4g^2}\right)^{-2}\right]. \tag{54}$$

Meanwhile, the power gain is independent of the input signal, as

$$G = \left(\frac{w-\gamma_{\mathrm{eg}}}{w+\gamma_{\mathrm{eg}}} N + \frac{\kappa_{\mathrm{S}}\kappa_{\mathrm{c}}}{4g^2}\right)^2 \Big/ \left(\frac{w-\gamma_{\mathrm{eg}}}{w+\gamma_{\mathrm{eg}}} N - \frac{\kappa_{\mathrm{S}}\kappa_{\mathrm{c}}}{4g^2}\right)^2. \tag{55}$$

The output power depends linearly on the weak input signal in the amplifying region (dashed lines in Fig. 3d).

In the masing region, the approximate inversion for weak input signal $|s_{\mathrm{in}}|^2 << \frac{w+\gamma_{\mathrm{eg}}}{8}(\frac{w-\gamma_{\mathrm{eg}}}{w+\gamma_{\mathrm{eg}}} N - \frac{\kappa_{\mathrm{S}}\kappa_{\mathrm{c}}}{4g^2})$ is

$$S_z = \frac{\kappa_S\kappa_c}{4g^2}\left(1-2\sqrt{\frac{2|s_{in}|^2}{w+\gamma_{eg}}\left(\frac{w-\gamma_{eg}}{w+\gamma_{eg}}N-\frac{\kappa_S\kappa_c}{4g^2}\right)^{-1}}\right), \quad (56)$$

and the power gain depends on the input signal (nonlinear amplification), as

$$G = \left(\sqrt{\frac{w+\gamma_{eg}}{2|s_{in}|^2}\left(\frac{w-\gamma_{eg}}{w+\gamma_{eg}}N-\frac{\kappa_S\kappa_c}{4g^2}\right)}-1\right)^2. \quad (57)$$

The output power is independent of the weak input signal in the masing region (solid lines in Fig. 3d).

Supplementary Fig. 1 shows the inversion, the power gain, and the noise temperature as functions of pump rate for a weak input signal (P_{in}=1 fW) and a fixed microwave cavity Q factor (Q=5×10^4). As in Supplementary Note 1, the threshold pump power for microwave amplifying is about 2.7 W (at $w=\gamma_{eg}$=200 s^{-1}), the threshold pump power for masing is about 4.3 W (at $w=w_{th}\approx$316 s^{-1}). For pump below the threshold for population inversion ($w<\gamma_{eg}$), the system is in the absorbing region (G_{dB}<0 dB). For pump above the threshold for population inversion ($w>\gamma_{eg}$), the system works as a microwave amplifier until reaching the masing region ($w>w_{th}$), which shuts down when over-pumping occurs ($w>w_{max}$). The power gain significantly increases near the masing threshold and the noise temperature is at sub-Kelvin level.

Supplementary Fig. 2 shows the dependence of the power gain on the cavity Q factor, the pump rate, and the input signal power. Note that the amplifying region at weak pump rate (e.g., 200 $s^{-1}<w<$316 s^{-1} for Q=5×10^4) becomes narrower as cavity Q factor increases (Fig. 2, Fig. 3, and Supplementary Fig. 2c).

Supplementary Note 6. Sensitivity to external magnetic field and temperature noises

To estimate the magnetic field sensitivity, we consider a magnetic field noise δB, which induces a frequency noise of all the NV spins $\delta\omega_S = \gamma_{NV}\delta B$.

The input noise of the cavity can be expressed as $\hat{F}_c(t) = \sqrt{\kappa_c^{ex}}\delta\hat{s}_{in}(t) + \sqrt{\kappa_c^{vac}}\delta\hat{s}_{vac}(t)$, including the cavity internal noise $\delta\hat{s}_{vac}(t)$ and the interaction with the input-output channel $\delta\hat{s}_{in}(t)$. The noise operators satisfy the

commutation relations $[\delta\hat{s}_{\rm in}(t),\delta\hat{s}_{\rm in}^{\dagger}(t')]=[\delta\hat{s}_{\rm vac}(t),\delta\hat{s}_{\rm vac}^{\dagger}(t')]=\delta(t-t')$. Both the internal noise and the input-output channel noise contribute to the cavity photon decay, i.e., $\kappa_{\rm c}=\kappa_{\rm c}^{\rm ex}+\kappa_{\rm c}^{\rm vac}$. The output field noise is $\delta\hat{s}_{\rm out}(t)=\delta\hat{s}_{\rm in}(t)-\sqrt{\kappa_{\rm c}^{\rm ex}}\delta\hat{a}(t)$, and the output field is $\hat{s}_{\rm out}(t)=-\sqrt{\kappa_{\rm c}^{\rm ex}}\hat{a}(t)$, corresponding to output photon flux $\left\langle\hat{s}_{\rm out}^{\dagger}\hat{s}_{\rm out}\right\rangle=\kappa_{\rm c}^{\rm ex}n_{\rm c}$. With a small internal cavity loss ($\kappa_{\rm c}^{\rm vac}/\kappa_{\rm c}\to 0$), all the cavity decay results in output, and the output power is $P_{\rm out}\approx\hbar\omega_{\rm c}\cdot\kappa_{\rm c}n_{\rm c}$.

The noises limit the measurement sensitivity when the maser device is used as a sensor. The noise spectrum mainly includes the following four contributions: (i) the photon shot noise, (ii) the Schawlow-Townes linewidth, (iii) the magnetic field noise, and (iv) the resonator size (dimensional) and dielectric constant noises resulting from temperature fluctuation. The photon shot noise (i) is dominant in short-time measurements but negligible in long-time measurement as compared with the quantum diffusion (ii, iii, and iv), which determines the coherence time or equivalently the maser linewidth. When the magnetic field (temperature) noise is dominant in the quantum diffusion, the magnetic field (temperature) noise can be derived from the maser coherence time. When the magnetic field and temperature are well stabilized with negligible fluctuations, the maser coherence time and hence the sensitivity is fundamentally limited by the ST-linewidth (ii).

In order to calculate the magnetic field sensitivity limit, we consider the resonator with negligible temperature noise. The magnetic field sensitivity can be obtain from the noise spectral density of the output field [46], $S_{\rm out}(\Omega)=\left\langle\left\{-i[\delta\hat{s}_{\rm out}(\Omega)-\delta\hat{s}_{\rm out}^{\dagger}(\Omega)]\right\}\left\{-i[\delta\hat{s}_{\rm out}(-\Omega)-\delta\hat{s}_{\rm out}^{\dagger}(-\Omega)]\right\}\right\rangle$, which gives

$$S_{\rm out}(\Omega)=1+\frac{4\kappa_{\rm c}n_{\rm c}}{\Omega^2}\left[\frac{\left(\frac{\kappa_{\rm S}}{2}\right)^2}{\left(\frac{\kappa_{\rm c}+\kappa_{\rm S}}{2}\right)^2+\Omega^2}n_{\rm incoh}\frac{\kappa_{\rm c}}{2n_{\rm c}}+\frac{\left(\frac{\kappa_{\rm c}}{2}\right)^2}{\left(\frac{\kappa_{\rm c}+\kappa_{\rm S}}{2}\right)^2+\Omega^2}(\gamma_{\rm NV}\delta B\sqrt{\tau})^2\right], \quad (58)$$

where the term "1" comes from photon shot noise, the first term in the bracket corresponds to background due to the maser phase fluctuation (linewidth), and the second term in the bracket results from the magnetic field noise. Thus the magnetic field sensitivity is limited by the other two terms as

$$\delta B\sqrt{\tau} = \frac{1}{\gamma_{\mathrm{NV}}}\frac{\kappa_{\mathrm{c}}+\kappa_{\mathrm{S}}}{\kappa_{\mathrm{c}}}\sqrt{\frac{\Omega^2}{4\kappa_{\mathrm{c}}n_{\mathrm{c}}}\left[1+\frac{4\Omega^2}{(\kappa_{\mathrm{c}}+\kappa_{\mathrm{S}})^2}\right]+\gamma_{\mathrm{ST}}}\,. \tag{59}$$

The photon shot noise term is negligible under the slow-noise condition $\Omega << \sqrt{2n_{\mathrm{incoh}}}\,\kappa_{\mathrm{c}}\kappa_{\mathrm{S}}/(\kappa_{\mathrm{c}}+\kappa_{\mathrm{S}}),\ (\kappa_{\mathrm{c}}+\kappa_{\mathrm{S}})/2$. So the magnetic field sensitivity is determined by coherence time (quantum diffusion) of the maser

$$\delta B\sqrt{\tau} = \frac{1}{\gamma_{\mathrm{NV}}}\frac{\kappa_{\mathrm{S}}}{\kappa_{\mathrm{c}}}\sqrt{n_{\mathrm{incoh}}\frac{\kappa_{\mathrm{c}}}{2n_{\mathrm{c}}}} = \frac{1}{\gamma_{\mathrm{NV}}}\frac{\kappa_{\mathrm{c}}+\kappa_{\mathrm{S}}}{\kappa_{\mathrm{c}}}\sqrt{\gamma_{\mathrm{ST}}} = \frac{1}{\gamma_{\mathrm{NV}}}\frac{\kappa_{\mathrm{c}}+\kappa_{\mathrm{S}}}{\kappa_{\mathrm{c}}}\sqrt{\frac{2}{T_{\mathrm{coh}}}}. \tag{60}$$

Similarly, the sensitivity limit of the temperature noise can be determined by setting the magnetic field as well stabilized. The result of the output noise is

$$S_{\mathrm{out}}(\Omega) = 1+\frac{4\kappa_{\mathrm{c}}n_{\mathrm{c}}}{\Omega^2}\left[\frac{(\kappa_{\mathrm{S}}/2)^2}{\left(\frac{\kappa_{\mathrm{S}}+\kappa_{\mathrm{c}}}{2}\right)^2+\Omega^2}n_{\mathrm{incoh}}\frac{\kappa_{\mathrm{c}}}{2n_{\mathrm{c}}}+\frac{(\kappa_{\mathrm{S}}/2)^2+\Omega^2}{\left(\frac{\kappa_{\mathrm{S}}+\kappa_{\mathrm{c}}}{2}\right)^2+\Omega^2}(g_0\delta T\sqrt{\tau})^2\right], \tag{61}$$

where $g_0 \approx (\alpha+\beta/2)\omega_{\mathrm{c}} = 165\ \mathrm{kHz\ K^{-1}}$ from equation (7). For low frequency noise [$\Omega << \sqrt{2n_{\mathrm{incoh}}}\,\kappa_{\mathrm{c}}\kappa_{\mathrm{S}}/(\kappa_{\mathrm{c}}+\kappa_{\mathrm{S}}),\ \kappa_{\mathrm{S}}/2$], the temperature sensitivity limited by the coherence time (quantum diffusion) is

$$\delta T\sqrt{\tau} = \frac{1}{g_0}\sqrt{n_{\mathrm{incoh}}\frac{\kappa_{\mathrm{c}}}{2n_{\mathrm{c}}}} = \frac{1}{g_0}\frac{\kappa_{\mathrm{c}}+\kappa_{\mathrm{S}}}{\kappa_{\mathrm{S}}}\sqrt{\gamma_{\mathrm{ST}}} = \frac{1}{g_0}\frac{\kappa_{\mathrm{c}}+\kappa_{\mathrm{S}}}{\kappa_{\mathrm{S}}}\sqrt{\frac{2}{T}} \tag{62}$$

Supplementary References

1. Grezes, C. *et al.* Multimode storage and retrieval of microwave fields in a spin ensemble. *Phys. Rev. X* **4**, 021049 (2014).
2. Clevenson, H. *et al*. Broadband magnetometry and temperature sensing with a light-trapping diamond waveguide. *Nat. Phys.* **11**, 393–397 (2015).
3. Wolf, T. *et al.* A subpicotesla diamond magnetometer. http://arxiv.org/abs/1411.6553 (2014).
4. Lesik, M. *et al.* Perfect preferential orientation of nitrogen-vacancy defects in a synthetic diamond sample. *Appl. Phys. Lett.* **104**, 113107 (2014).
5. Michl, J. *et al*. Perfect alignment and preferential orientation of nitrogen-vacancy centers during chemical vapor deposition diamond growth on (111) surfaces. *Appl. Phys. Lett*. **104**, 102407 (2014).
6. Fukui, T. *et al.* Perfect selective alignment of nitrogen-vacancy centers in diamond. *Appl. Phys. Express* **7**, 055201 (2014).
7. Acosta, V. M. *et al.* Diamonds with a high density of nitrogen-vacancy centers for magnetometry applications. *Phys. Rev. B* **80**, 115202 (2009).
8. Acosta, V. M. *et al.* Broadband magnetometry by infrared-absorption detection of nitrogen-vacancy ensembles in diamond. *Appl. Phys. Lett.* **97**, 174104 (2010).
9. Ishikawa, T. *et al.* Optical and spin coherence properties of nitrogen-vacancy centers placed in a 100 nm thick isotopically purified diamond layer. *Nano. Lett.* **12**, 2083–2087 (2012).
10. Ohashi, K. *et al.* Negatively charged nitrogen-vacancy centers in a 5 nm thin ^{12}C diamond film. *Nano. Lett.* **13**, 4733–4738 (2013).
11. Balasubramanian, G. *et al.* Ultralong spin coherence time in isotopically engineered diamond. *Nat. Mater.* **8**, 383–387 (2009).
12. Mizuochi, N. *et al.* Coherence of single spins coupled to a nuclear spin bath of varying density. *Phys. Rev. B* **80**, 041201(R) (2009).
13. Kubo, Y. *et al.* Hybrid quantum circuit with a superconducting qubit coupled to a spin ensemble. *Phys. Rev. Lett.* **107**, 220501 (2011).
14. Redman, D. A., Brown, S., Sands, R. H. & Rand, S. C. Spin dynamics and electronic states of N-V centers in diamond by EPR and four-wave-mixing spectroscopy. *Phys. Rev. Lett.* **67**, 3420–3423 (1991).

15. Takahashi, S., Hanson, R., van Tol, J., Sherwin, M. S. & Awschalom, D. D. Quenching spin decoherence in diamond through spin bath polarization. *Phys. Rev. Lett.* **101**, 047601 (2008).
16. Jarmola, A., Acosta, V. M., Jensen, K., Chemerisov, S. & Budker, D. Temperature- and magnetic-field-dependent longitudinal spin relaxation in nitrogen-vacancy ensembles in diamond. *Phys. Rev. Lett.* **108**, 197601 (2012).
17. Sebastian, M. T. *Dielectric Materials for Wireless Communication* (Elsevier, 2008).
18. Krupka, J., Derzakowski, K., Tobar, M., Hartnett, J. & Geyer, R. G. Complex permittivity of some ultralow loss dielectric crystals at cryogenic temperatures. *Meas. Sci. Technol.* **10**, 387–392 (1999).
19. le Floch, J.-M., Tobar, M. E., Cros, D. & Krupka, J. Low-loss materials for high Q-factor Bragg reflector resonators. *Appl. Phys. Lett.* **92**, 032901 (2008).
20. Oxborrow, M., Breeze, J. D. & Alford, N. M. Room-temperature solid-state maser. *Nature* **488**, 353–356 (2012).
21. Petosa, A. *Dielectric Resonator Antenna Handbook* (Artech House, 2007).
22. Raich, H. & Blümler, P. Design and construction of a dipolar Halbach array with a homogeneous field from identical bar magnets: NMR Mandhalas. *Concept Magn. Reson. B: Magn. Reson. Eng.* **23B**, 16–25 (2004).
23. Anferova, S. *et al.* A mobile NMR device for measurements of porosity and pore size distributions of drilled core samples. *Concept Magn. Reson. B: Magn. Reson. Eng.* **23B**, 26–32 (2004).
24. Doğan, N., Topkaya, R., Subaşi, H., Yerli, Y. & Rameev, B. Development of Halbach magnet for portable NMR device. *J. Phys.: Conf. Ser.* **153**, 012047 (2009).
25. Kajfez, D. & Guillon, P. (Eds.) *Dielectric Resonators* (Artech House, 1986).
26. Chen, L. F., Ong, C. K., Neo, C. P., Varadan, V. V. & Varadan V. K. *Microwave Electronics: Measurement and Materials Characterization* (Wiley, 2004).
27. Probst, S. *et al*. Three-dimensional cavity quantum electrodynamics with a rare-earth spin ensemble. *Phys. Rev. B* **90**, 100404R (2014).
28. Schuster, D. I. *et al.* High-cooperativity coupling of electron-spin ensembles to superconducting cavities. *Phys. Rev. Lett.* **105**, 140501 (2010).

29. Kubo,Y. *et al.* Strong coupling of a spin ensemble to a superconducting resonator. *Phys. Rev. Lett.* **105**, 140502 (2010).
30. Abe, E., Wu, H., Ardavan, A. & Morton, J. J. L. Electron spin ensemble strongly coupled to a three-dimensional microwave cavity. *Appl. Phys. Lett.* **98**, 251108 (2011).
31. Dobrovinskaya, E. R., Lytvynov, L. A. & Pishchik, V. *Sapphire: Material, Manufacturing, Applications* (Springer, 2009).
32. Tobar, M. E., Krupka, J., Hartnett, J. G., Ivanov, E. N. & Woode, R. A. Sapphire-rutile frequency-temperature compensated whispering gallery microwave resonators. *Proc. IEEE Int. Freq. Contr. Symp.* 1000–1008 (1997).
33. Acosta, V. M. *et al.* Temperature dependence of the nitrogen-vacancy magnetic resonance in diamond. *Phys. Rev. Lett.* **104**, 070801 (2010).
34. Acosta, V. M., Jarmola, A., Bauch, E. & Budker, D. Optical properties of the nitrogen-vacancy singlet levels in diamond. *Phys. Rev. B* **82**, 201202 (2010).
35. Robledo, L., Bernien, H., van der Sar, T. & Hanson, R. Spin dynamics in the optical cycle of single nitrogen-vacancy centres in diamond. *New J. Phys.* **13** 025013 (2011).
36. Tetienne, J.-P. *et al.* Magnetic-field-dependent photodynamics of single NV defects in diamond: an application to qualitative all-optical magnetic imaging. *New J. Phys.* **14**, 103033 (2012).
37. Manson, N. B., Harrison, J. P. & Sellars, M. J. Nitrogen-vacancy center in diamond: Model of the electronic structure and associated dynamics. *Phys. Rev. B* **74**, 104303 (2006).
38. Neumann, P. *et al.* Excited-state spectroscopy of single NV defects in diamond using optically detected magnetic resonance. *New J. Phys.* **11**, 013017 (2009).
39. Wrachtrup, J. Defect center room-temperature quantum processors. *Proc. Natl. Acad. Sci. U.S.A.* **107**, 9479–9480 (2010).
40. Wee, T. L. *et al*. Two-photon excited fluorescence of nitrogen-vacancy centers in proton-irradiated type Ib diamond. *J. Phys. Chem. A* **111**, 9379–9386 (2007).
41. Wang, Z.-H. & Takahashi, S. Spin decoherence and electron spin bath noise of a nitrogen-vacancy center in diamond. *Phys. Rev. B* **87**, 115122 (2013).

42. Kolobov, M. I., Davidovich, L., Giacobino, E. & Fabre, C. Role of pumping statistics and dynamics of atomic polarization in quantum fluctuations of laser sources. *Phys. Rev. A* **47**, 1431–1446 (1993).
43. Bohnet, J. G., Chen, Z., Weiner, J. M., Cox, K. C. & Thompson, J. K. Relaxation oscillations, stability, and cavity feedback in a superradiant Raman laser. *Phys. Rev. Lett*. **109**, 253602 (2012).
44. Meiser, D., Ye, J., Carlson, D. R. & Holland, M. J. Prospects for a millihertz-linewidth laser. *Phys. Rev. Lett.* **102**, 163601 (2009).
45. Meiser, D. & Holland, M. J. Steady-state superradiance with alkaline-earth-metal atoms. *Phys. Rev. A* **81**, 033847 (2010).
46. Weiner, J. M., Cox, K. C., Bohnet, J. G., Chen, Z. & Thompson, J. K. Superradiant Raman laser magnetometer. *Appl. Phys. Lett.* **101**, 261107 (2012).
